# *Salmonella* Typhi asparaginase-dependent activation of GCN2 promotes bacterial killing in murine macrophages

**DOI:** 10.1128/iai.00178-26

**Published:** 2026-06-15

**Authors:** Zachary M. Powers, Michael J. McFadden, Gi Young Lee, Tracey Schultz, Luiza A. Castro Jorge, Daniel F. Edwards, Simon Sanchez-Paiva, Jonathan Z. Sexton, Katherine R. Spindler, Jeongmin Song, Mary X. O'Riordan

**Affiliations:** 1 Department of Microbiology and Immunology, University of Michigan Medical School 12266 , Ann Arbor, Michigan, USA; 2 Department of Microbiology and Immunology, Cornell University College of Veterinary Medicine 43317, Ithaca, New York, USA; 3 Department of Molecular Biology, Jeonbuk National University 26714, Jeonju, Jeonbuk State, Republic of Korea; 4 Department of Internal Medicine, Division of Gastroenterology, University of Michigan Medical School 12266 , Ann Arbor, Michigan, USA; 5 Department of Medicinal Chemistry, College of Pharmacy, University of Michigan 15514, Ann Arbor, Michigan, USA; Stanford University School of Medicine, Stanford, California, USA

**Keywords:** innate immunity, gram-negative bacteria, nutritional immunity

## Abstract

Many intracellular pathogens stimulate host cell stress by directly or indirectly causing an imbalance in host nutrients. Depletion of amino acid pools, in particular, can act as a danger signal to infected cells. Using a restrictive host model of *Salmonella enterica* serovar Typhi (*S*. Typhi) infection, we identify early induction of the integrated stress response (ISR) by viable bacteria, but not by heat-killed bacteria. Genetic deletion of the amino acid-sensing ISR kinase GCN2 (also known as EIF2AK4) prevented early ISR activation during *S*. Typhi infection and murine macrophages lacking GCN2 show impaired bacterial clearance and decreased cytokine output. Supplementation of wild-type C57BL/6 murine macrophages with only the non-essential amino acid asparagine was sufficient to suppress *S*. Typhi-induced ISR activation, and deletion of *S*. Typhi *ansB*, encoding an asparaginase, prevented ISR activation during infection. Pharmacological inhibition of mammalian target of rapamycin (mTOR), the other major amino acid-sensing pathway in eukaryotic cells, prevented GCN2 activation and ISR induction in murine macrophages, indicating an upstream role for mTOR in signaling to GCN2. These findings suggest a role for the ISR in macrophage innate immune responses to *S*. Typhi infection and highlight a potential difference in nutrient-dependent signaling between the *S*. Typhi-susceptible human host and the restrictive murine host centered around asparagine, mTOR, and GCN2.

## INTRODUCTION

Despite an estimated 11–21 million annual cases of typhoidal *Salmonella* worldwide, specific research on *Salmonella enterica* serovar Typhi (*S*. Typhi) remains relatively constrained due to its narrow host range (1). Productive *S*. Typhi infection largely occurs only in the human host, causing systemic typhoid fever, while commonly used murine models do not support infection, leading to the development of the closely related *Salmonella enterica* serovar Typhimurium (*S*. Typhimurium) as a more tractable model to study typhoid fever in mice (2, 3). Therefore, murine macrophages can be investigated as a restrictive host model of *S*. Typhi infection to uncover mechanisms that successfully control this pathogen (4, 5). Macrophages are early sentinels of infection, and nutrient availability is central in shaping macrophage innate immune function (6–8). For example, host depletion of tryptophan by indoleamine dioxygenase during *Coxiella burnetti* infection limits bacterial replication (9). Lactate derived from phagocytized bacteria can trigger neutrophil NETosis (10), highlighting how fluctuations in nutrient pools play a key role in alerting the host to infection as an indirect mechanism of pathogen sensing.

Mammalian cells use two primary systems to sense amino acid depletion: mammalian target of rapamycin (mTOR) and the integrated stress response (ISR). Well studied in the context of sterile disease such as cancers, the ISR has a variable role in the immune response to bacterial, viral, and parasitic infections (11–13). Upon activation of any of the four ISR stress-sensing kinases (PERK, GCN2, HRI, and PKR), general cellular translation is reduced through phosphorylation of eIF2α, which allows for preferential translation of *Atf4* mRNA, a transcription factor that generally functions to return the cell to homeostasis or trigger stress-induced cell death (14). Of the four ISR kinases, GCN2 acts as a sensor for amino acid depletion primarily in response to uncharged tRNAs (15, 16). Both Gram-positive and Gram-negative bacterial pathogens induce the ISR during infection (11, 17–20). However, pathogen-induced ISR activation mechanisms and outcomes differ by pathogen, cell type, stimulating signal, and environmental cues, suggesting that the specific context of ISR activation influences infection outcomes (21, 22).

Extensive studies establish mTOR as a prominent regulator of cellular metabolism; however, recent findings have revealed new aspects of mTOR signaling and function particularly when associated with lysosomes, a major interface for nutrient acquisition (23–26). mTOR forms distinct complexes, termed mTORC1 (mTOR complex 1) or mTORC2, defined by interacting partners in the complex (27). mTORC1 at the cytosolic-facing lysosomal membrane senses nutrient repletion, promoting growth and inhibiting autophagy (28). mTORC2 activation may occur due to nutrient depletion, repletion, or metabolic waste, with sub-populations localized to the plasma membrane, mitochondria, and endosomes (29, 30). Prior studies demonstrate that mTOR and the ISR can signal bidirectionally in a context-dependent manner, with both pathways capable of regulating transcription, translation, and cellular nutrient pools (31).

As a facultative intracellular pathogen, *Salmonella enterica* activates both intra- and extracellular pathogen-sensing pathways while perturbing host defenses through effector secretion (32–34). As first responders to early *Salmonella* infection, host macrophages can be co-opted by *Salmonella*, where the bacteria survive inside *Salmonella*-containing vacuoles (SCVs) and may disseminate to the spleen, liver, and gallbladder through macrophage-dependent and -independent mechanisms (35). Prior studies aimed at identifying human macrophage host factors critical for modulating *Salmonella* infection through a genome-wide CRISPR screen suggested a role for the ISR kinase PKR (36). Together with a prior report that nutrient depletion triggered the ISR during *S*. Typhimurium infection of HeLa cells (11), these findings led us to investigate how host amino acid sensors and the responsive signaling pathways sculpt the macrophage innate immune response during early *S*. Typhi infection of a restrictive host cell.

## RESULTS

### The ISR is induced by live *S*. Typhi during murine macrophage infection

**Fig 1:**
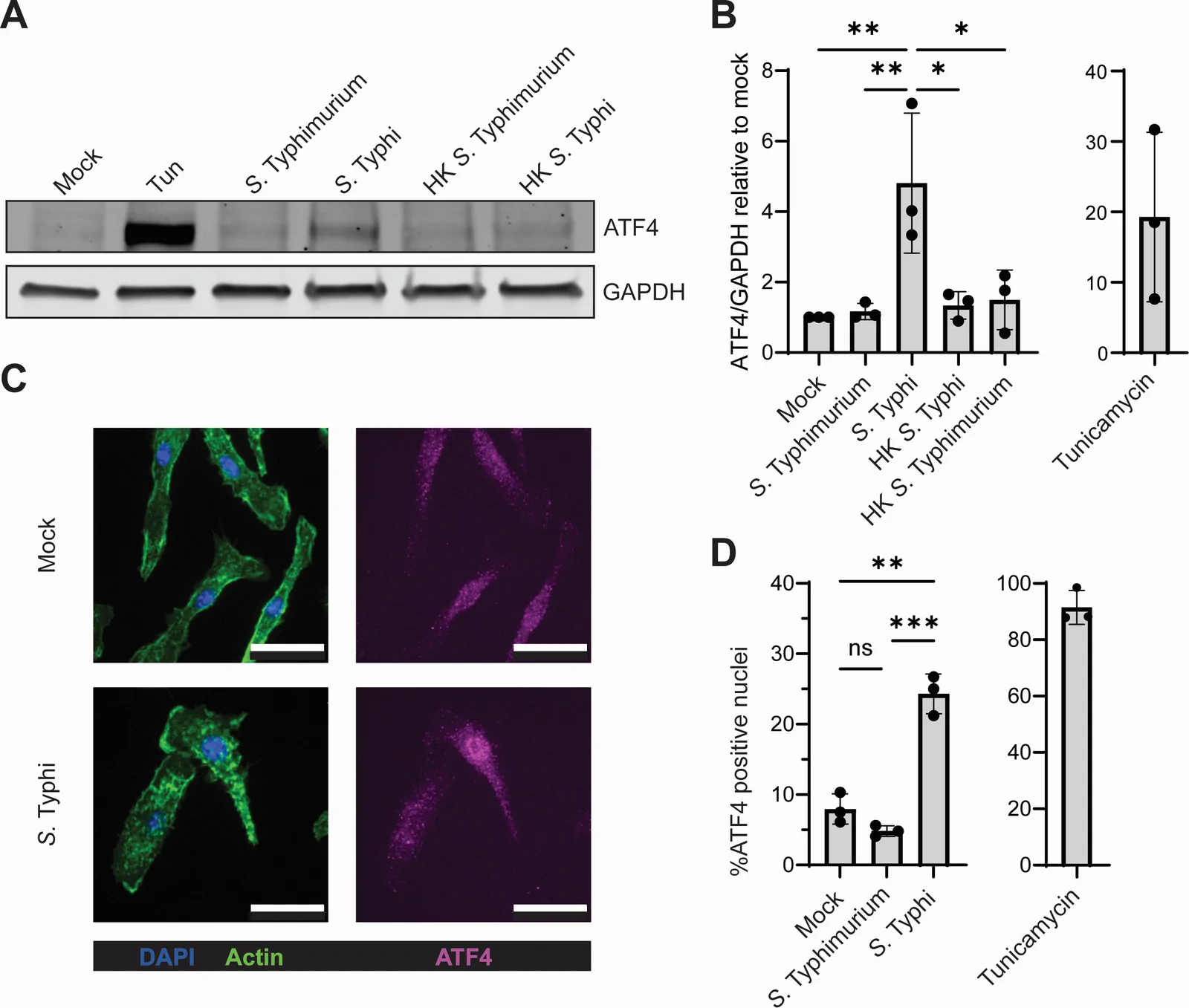
Live *S*. Typhi activates the integrated stress response during murine macrophage infection. WT BMDMs infected with MOI 10 *S*. Typhimurium or *S*. Typhi, heat-killed (HK) equivalents, or treated with ISR-positive control tunicamycin (tun; 10 µM) for 8 h. (**A**) Whole-cell lysates were analyzed by immunoblot for ATF4 as a readout of ISR induction. A representative immunoblot is shown, and three experimental replicates were quantified by densitometry and graphed in **panel B**. (**C**) Representative immunofluorescence microscopy of mock and S. Typhi-infected BMDMs at 8 hpi is shown as a composite image for DNA (DAPI, blue) and F-actin (phalloidin, green), and a separate image for ATF4 (magenta). Scale bar = 25 µm. (**D**) Means for ATF4-positive nuclei derived from three independent experimental replicates as described in **panel C**. Each point represents the mean of > 30 nuclei per replicate sample. Unpaired one-way ANOVA and Tukey’s post-test were used to compare column means with multiple comparisons. Error bars represent SD. *P*-value: *<0.05; **<0.01; ***<0.001.

Bacterial infection activates the ISR in a context-dependent way in different host cell types, so we first tested if the ISR was induced during infection of primary murine bone marrow macrophages (BMDMs) by *S. enterica* serovars. Macrophages were harvested by whole-cell lysis and analyzed by immunoblot for ATF4 protein, which represents the convergence point of the ISR (11, 17–20). Using non-typhoidal *S*. Typhimurium and typhoidal *S*. Typhi infection of BMDMs, we observed that *S*. Typhi, but not *S*. Typhimurium, infection increased ATF4 levels (Fig. 1A and B). Tunicamycin (tun) was used as a positive control for ISR activation as it robustly induces the ISR through endoplasmic reticulum stress via the sensor kinase PERK (21). Treatment of BMDMs with heat-killed bacterial equivalents did not result in increased ATF4 (Fig. 1A and B), suggesting an active role for *S*. Typhi in activating the ISR. To orthogonally test this observation, we infected BMDMs with *Salmonella* and measured the number of cells with ATF4-positive nuclei using quantitative immunofluorescence microscopy. Consistent with bulk ATF4 induction observed by immunoblot, we found that only *S*. Typhi-infected samples showed ISR induction (Fig. 1C and D). These results demonstrate that despite *S*. Typhi and *S*. Typhimurium sharing 89% genetic similarity, these two distinct serovars of *Salmonella enterica* differentially engage the ISR in murine macrophages, allowing us to explore the role that this stress response pathway may play in innate immune responses to *S*. Typhi infection in the context of a restrictive host (37).

### *Salmonella* Typhi-driven macrophage ISR activation requires GCN2

**Fig 2:**
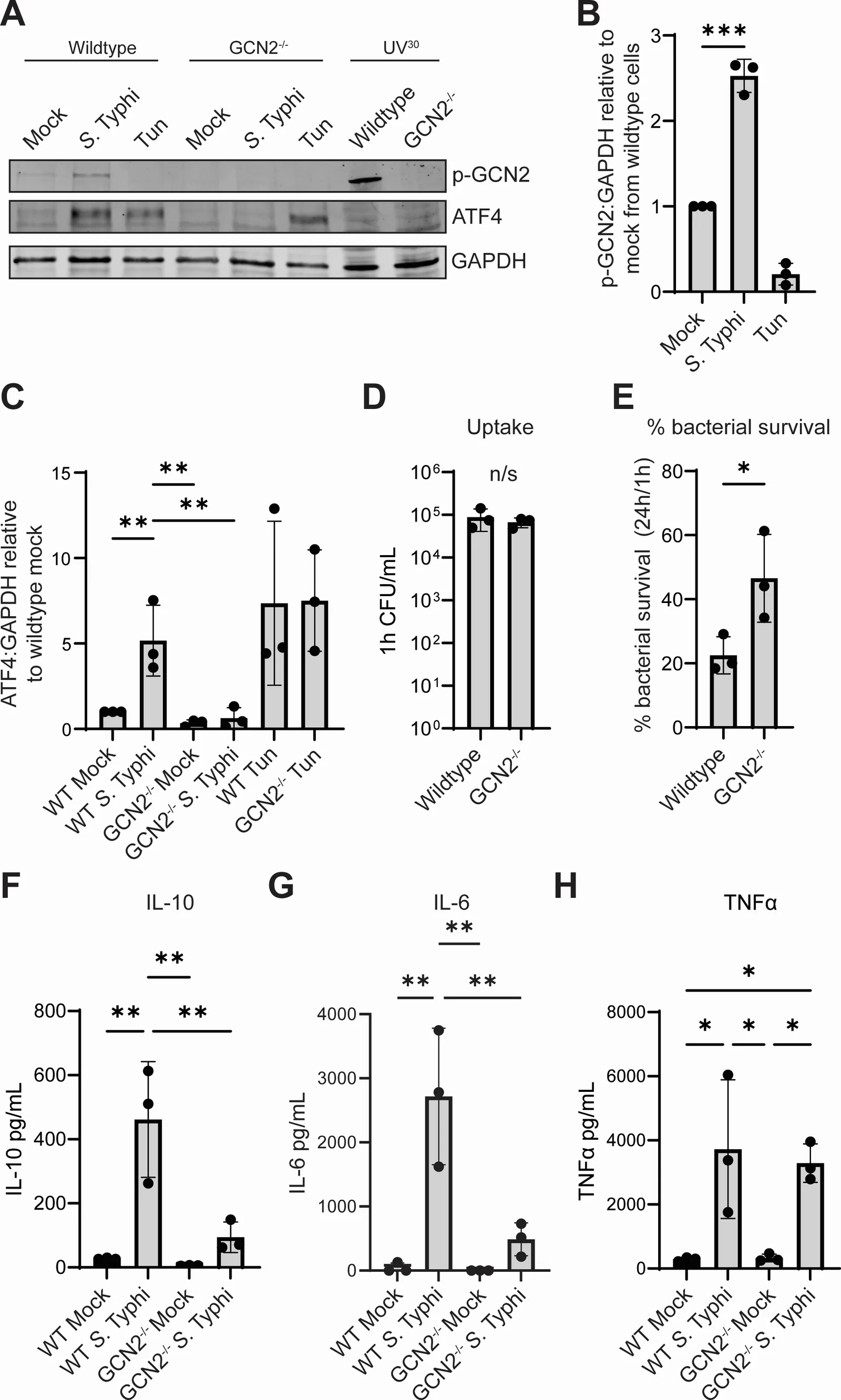
GCN2 is required for *S*. Typhi-driven macrophage ISR activation in murine macrophages. (**A–C**) WT and *Gcn2^−/^*^−^ BMDMs infected with MOI 10 *S*. Typhi for 8 h. Whole-cell lysates from ultraviolet (UV)-treated BMDM exposed to 500 J/m^2^ and recovered for 30 min were used as a positive control for phospho-GCN2. (**A**) Representative immunoblot of whole-cell lysates subjected to SDS-PAGE and probed with antibodies against phospho-GCN2 and ATF4. (**B**) Densitometry quantification of phospho-GCN2 and (**C**) ATF4 from three experimental replicates of (**A**). Tunicamycin-treated conditions were excluded from analysis. (**D–H**) WT and *Gcn2^−/^*^−^ BMDMs were infected with MOI 10 *S*. Typhi, then lysed and plated for colony-forming units to measure (**D**) bacterial uptake at 1 hpi and (**E**) 24 h bacterial survival (relative to 1 hpi). (**F–H**) Supernatants from WT and *Gcn2^−/^*^−^ BMDMs infected with MOI 10 *S*. Typhi were collected at 8 hpi and assayed by ELISA for IL-6, IL-10, and TNFα. (**B, D, E**) Unpaired two-tailed *t*-test. (**C, F–H**) Unpaired one-way ANOVA and Tukey’s post-test were used to compare column means with multiple comparisons. Error bars represent SD. *P*-value: *<0.05; **<0.01; ***<0.001.

We sought to identify the eIF2α kinase responsible for ISR activation during *S*. Typhi infection of BMDMs, first testing the eIF2α kinase GCN2 (also known as EIF2AK4). Guiding us were reports of two closely related Gram-negative bacteria, adherent-invasive *Escherichia coli* and *S*. Typhimurium, triggering GCN2 during *ex vivo* infection of transformed human epithelial cells (11, 19, 38). In mammalian cells, GCN2 is canonically activated by amino acid starvation, primarily sensing increased levels of uncharged tRNAs and resulting in GCN2 phosphorylation (15, 16). Non-canonical GCN2 activation may result from ribosomal damage or UV-induced ribosomal collisions, although non-canonical activation does not necessarily induce ATF4 (39–41). We tested the necessity of GCN2 for ATF4 induction during *S*. Typhi infection of wild-type (WT) and GCN2-deficient (*Gcn2^−/^*^−^) BMDMs. Immunoblotting with a phospho-specific antibody that recognizes the activating GCN2 phosphorylation site revealed that *S*. Typhi induced p-GCN2 in infected conditions, and only WT BMDMs exhibited phosphorylated GCN2 upon UV treatment as a positive control (Fig. 2A and B). Additionally, *S*. Typhi-infected *Gcn2^−/^*^−^ BMDMs did not increase ATF4 levels in response to *S*. Typhi but were still capable of ISR activation when treated with tunicamycin, suggesting that other ISR components remained functional (Fig. 2A and C). We conclude from these observations that GCN2 is the eIF2α kinase required for ISR induction by BMDMs in the early response to *S*. Typhi infection.

GCN2 was previously hypothesized to regulate *S*. Typhimurium survival in HeLa cells, but its requirement has not been tested directly there or in macrophages (11). Macrophages act early in the innate immune response to phagocytize and kill invading bacteria while signaling to other cells via cytokine secretion. In permissive host-serovar matches, *Salmonella* hijack macrophages and disseminate to distal sites, such as the spleen, liver, bone marrow, and gallbladder (42–46). While macrophages generally are not proliferative environments for *Salmonella*, a subset of infected macrophages support bacterial replication, which influences macrophage functional phenotypes (47–49). We tested whether GCN2 was required for *S*. Typhi restriction in WT and *Gcn2^−/^*^−^ BMDMs and found significant differences in the survival of *S*. Typhi after 24 h of infection (Fig. 2D and E). We infected *Gcn2^−/^*^−^ and WT C57BL/6 animals with *S*. Typhi, as well as *Gcn2^−/−^Pkr^−/^*^−^ double-knockout mice to test for potential redundancy in activating the ISR, but did not observe any significant differences in susceptibility by 24 h post-infection, when we could still recover *S*. Typhi (Fig. S1A, B and F through H), potentially indicating more complex interactions *in vivo*. While not statistically significant, levels of circulating cytokines IL-6, IL-10, and TNFα in *Gcn2^−/−^Pkr^−/^*^−^ double-knockout mice trended lower during *S*. Typhi infection relative to cytokine levels in WT mice (Fig. S1I through K). To determine if GCN2 was also activated by *S*. Typhi infection in human cells, we infected U-937 human monocyte cells differentiated with phorbol myristate acetate (PMA). Differentiated U-937 cells did not activate GCN2 during *S*. Typhi or Typhimurium infection, highlighting context-dependent GCN2 activation as a potential contributing factor distinguishing *S*. Typhi infection in human vs mouse cells. Tunicamycin treatment increased ATF4, showing that ISR induction remained intact (Fig. S2). Treatment of U-937 cells with the GCN2 activator halofuginone did not reduce bacterial burdens after 24 h, indicating that GCN2 activation alone is insufficient for bacterial restriction in these human cells (Fig. S3). Finally, we returned to murine macrophages to test if GCN2 was required for cytokine production during *S*. Typhi infection. We assayed supernatants from infected BMDMs by ELISA and found that IL-6 and IL-10, but not TNFα, were reduced in the absence of GCN2 (Fig. 2F through H). Together, these results are consistent with a model where GCN2 enhances the murine macrophage innate immune response to *S*. Typhi by augmenting infection control and potentiating early cytokine production.

### ISR activation can be suppressed by addition of L-asparagine during *S*. Typhi infection

**Fig 3:**
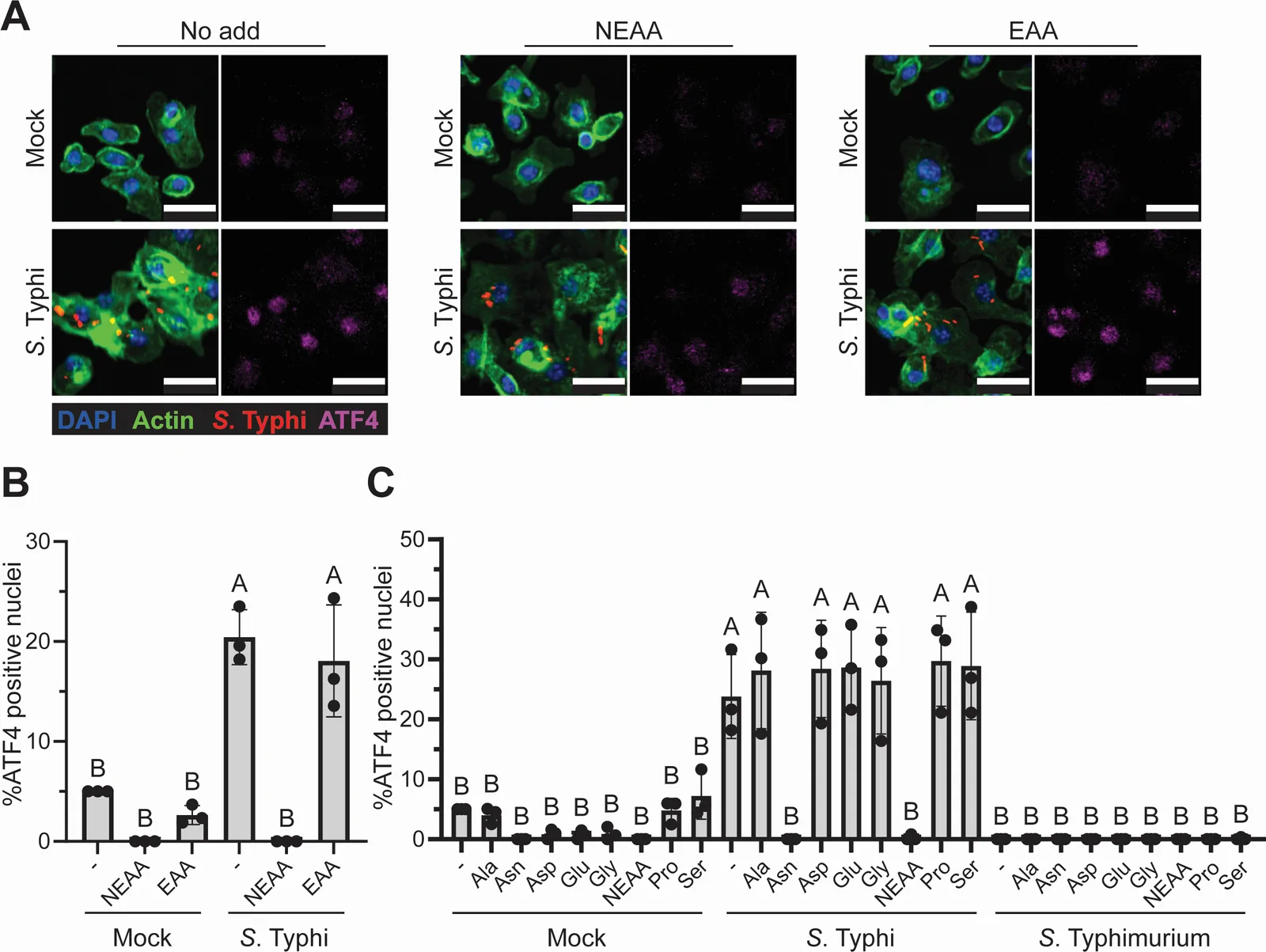
L-asparagine supplementation suppresses *S*. Typhi-induced ISR activation in murine macrophages. (**A and B**) WT BMDMs infected with MOI 10 *S*. Typhi constitutively expressing a DsRed fluorescent reporter were treated with grouped essential- or non-essential amino acids (EAA/NEAA) for 8 h. (**A**) Representative immunofluorescence microscopy of ATF4-positive nuclei with composite image including DNA (DAPI, blue), F-actin (phalloidin, green), and *S*. Typhi (DsRed fluorescent protein expression, red), and a separate ATF4 (magenta) image. Scale bar = 25 µm. (**B**) Quantification of three experimental replicates of panel **A**, with points representing means of *n* = 3 experiments with >1,000 nuclei per condition per replicate. (**C**) WT BMDMs infected with MOI 10 *S*. Typhi were treated with indicated individual NEAAs during 8 h of infection. Quantification of ATF4-positive nuclei in *S*. Typhi-infected BMDMs treated with individual NEAAs, measured by immunofluorescence microscopy. Points represent means of *n* = 3 experiments with >1,000 nuclei per condition per replicate. (**B and C**) Unpaired one-way ANOVA and Tukey’s post-test were used to compare column means, with compact letter display for statistical representation of comparisons with *P*-value < 0.05 (51). For compact letter display, two groups marked with the same letter are not statistically different, whereas two groups marked with different letters are statistically different. Error bars represent SD.

Having established GCN2 as the primary eIF2α kinase driving early ISR induction during *S*. Typhi infection, we next aimed to determine the signal activating GCN2. Since GCN2 is canonically activated in response to uncharged tRNAs in affected cells and infection may alter macrophage amino acid abundance, we supplemented amino acids into *S*. Typhi-infected BMDM culture conditions (15, 16, 50). By supplementing with two groups of amino acids—essential and non-essential—we determined that supplementation with only non-essential amino acids (NEAAs) suppressed *S*. Typhi-induced ISR activation, as measured by ATF4 nuclear intensity using quantitative immunofluorescence microscopy (Fig. 3A and B; Table S1). Finding ISR suppression by NEAA supplementation during *S*. Typhi infection was surprising, since a previous report of *S*. Typhimurium GCN2 induction in HeLa cells was associated with low levels of the essential amino acids (EAAs) leucine and isoleucine (11). These contrasting findings highlight how the contextual differences between these closely related pathogens and their host environment manifest differently despite both triggering the ISR. To identify the specific amino acid(s) capable of suppressing ISR activation in macrophages, *S*. Typhi and S. Typhimurium BMDM infections were cultured with the addition of individual NEAAs (minus glutamine which was already provided in excess in our standard BMDM culture medium) (Table S2), and nuclear ATF4 levels were measured by quantitative immunofluorescence microscopy (Fig. 3C). No *S*. Typhimurium infection conditions showed any significant differences from mock treated. However, of all individual amino acids tested, only the addition of asparagine was sufficient to suppress the ISR in *S*. Typhi-infected macrophages, suggesting that *S*. Typhi-dependent depletion of the NEAA asparagine triggers ISR activation.

### The *S*. Typhi L-asparaginase II AnsB is required to induce the ISR during murine macrophage infection

**Fig 4:**
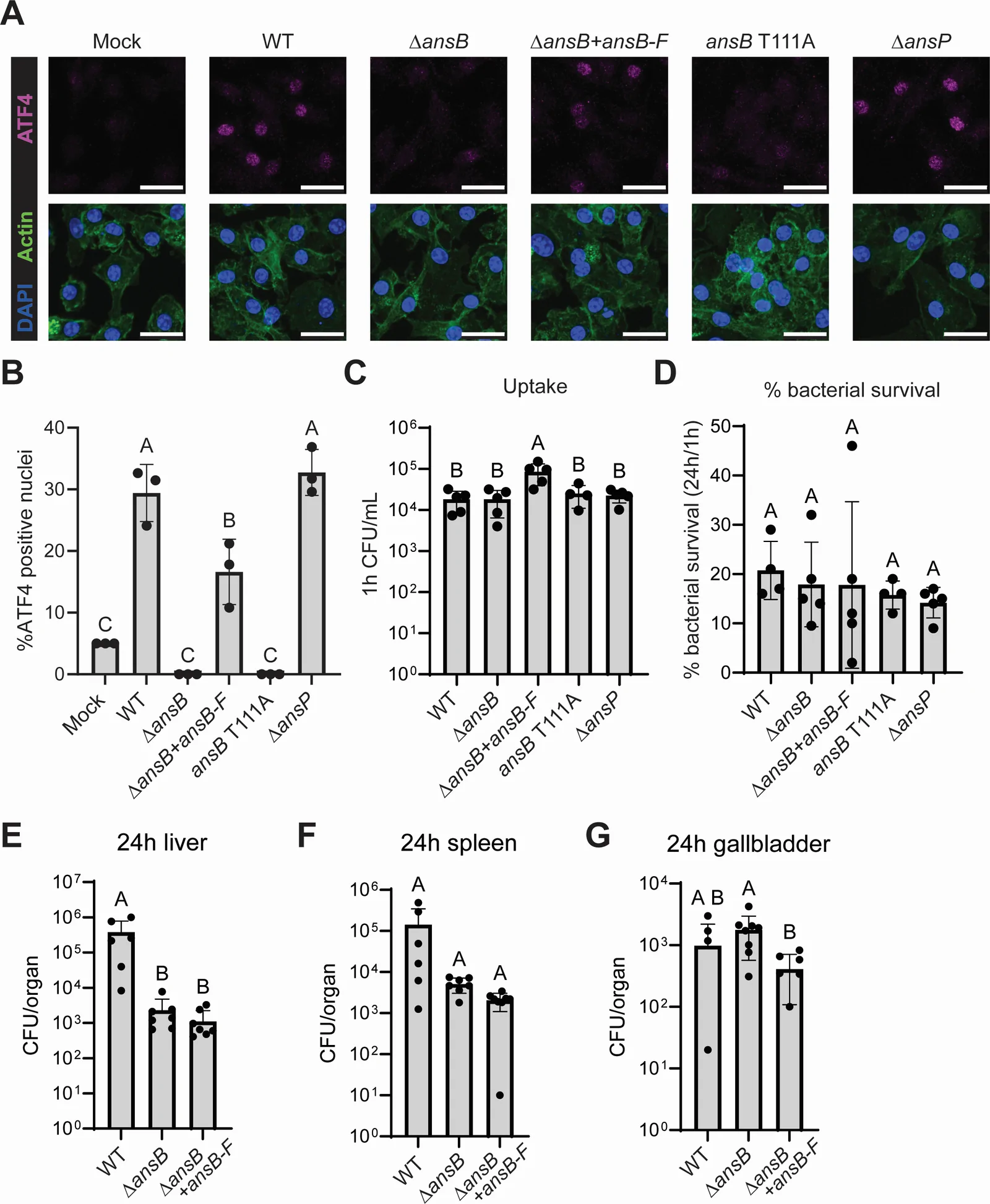
The *S*. Typhi L-asparaginase II AnsB is required for murine macrophage ISR activation during infection. (**A and B**) Murine BMDMs infected with MOI 10 with *S*. Typhi wild-type Ty2, L-asparaginase II (*ansB*) mutants, or asparagine transporter (*ansP*) were imaged at 8 hpi by automated confocal immunofluorescence microscopy. (**A**) Representative immunofluorescence microscopy of ATF4-positive nuclei with composite DNA (DAPI, blue) and F-actin (phalloidin, green), and a separate image showing ATF4 (magenta). Scale bar = 25 µm. (**B**) Quantification of three experimental replicates of panel **A** with points representing means of *n* = 3 experiments with >1,000 nuclei per condition per replicate. (**C and D**) Murine BMDMs infected with MOI 10 *S*. Typhi wild-type Ty2, *ansB,* or *ansP* mutants were lysed, and CFUs were enumerated to measure (**C**) bacterial uptake at 1 hpi and (**D**) bacterial survival at 24 hpi relative to 1 hpi. (**E–G**) WT C57BL/6 mice were infected intraperitoneally with 5 × 10^6^ CFU/animal of *S*. Typhi. At 24 hpi, the liver (**E**), spleen (**F**), and gallbladder (**G**) were harvested, homogenized, and CFU enumerated. (**B–G**) Unpaired one-way ANOVA and Tukey’s post-test were used to compare column means, with a compact letter display for statistical representation of comparisons with *P*-value < 0.05 (51). For the compact letter display, two groups marked with the same letter are not statistically different, whereas two groups marked with different letters are statistically different. Error bars represent SD.

*Salmonella* species encode an asparaginase enzyme, and we reasoned that bacterial L-asparaginase (ASNase) might contribute to ISR induction during macrophage infection. Of interest, recombinant ASNases are used as an anticancer treatment to restrict certain types of leukemic growth, and this treatment can activate GCN2 (52). ASNases catalyze the hydrolytic degradation of L-asparagine to aspartic acid and ammonia and are present in genomes across the tree of life, yet are not expressed in humans or mice outside of reproductive cells and organs (53–57). *S*. Typhimurium ASNase II, encoded by *ansB*, is expressed and modulates T cell immune function in a mouse model of infection (58–61). To test if the *S*. Typhi ASNase II is required for ISR induction in murine macrophages, we generated a collection of *S*. Typhi mutants: an *ansB* clean deletion (*ΔansB*), an *ansB* catalytic-dead point mutant (T111A), an *ansB* clean deletion with in-locus complementation with FLAG tag (*ΔansB + ansB*-*F*), and an asparagine transporter *ansP* clean deletion (*ΔansP*). Mutants grew similarly to WT *S*. Typhi in axenic culture, and expression in the complemented strain was experimentally confirmed (Fig. S4). We performed BMDM infections with these mutants and found that *ansB* deletion or T111A mutation was sufficient to prevent ISR induction during infection, as measured by ATF4 nuclear intensity (Fig. 4A and B). Consistent with Fig. 3, supplementation with Asn or NEAAs prevented ISR induction in the *ΔansB + ansB*-*F* and *ΔansP* infection conditions (Fig. S5). Native locus complementation with *ansB-F* restored ISR induction, although not to WT levels (Fig. 4B). These results suggest a model where depletion of asparagine (or an increase in its degradation products within the SCV) induces the ISR. Bacterial mutants were taken up at similar rates as WT *S*. Typhi by WT BMDMs, although *ΔansB + ansB-F* showed slightly higher uptake (Fig. 4C). While we had reasoned that *ΔansB* and *ansB* T111A mutant *S*. Typhi might exhibit increased survival due to reduced ISR activation, we instead found that WT and mutant *S*. Typhi survived similarly at 24 h post-infection relative to 1 h (Fig. 4C and D). We next infected C57BL/6 mice with wild-type and *ΔansB S*. Typhi for 24 h to test the requirement for *ansB* during *in vivo* infection and measured bacterial burden in the spleen, liver, and gallbladder at 24 hpi. *ΔansB S*. Typhi had lower liver burden compared to WT bacteria (Fig. 4E), and we observed a trend toward lower burden in the spleen (Fig. 4F), but did not find statistically significant differences in the gallbladder (Fig. 4G), an important reservoir for chronic infection (42). These results suggest that *ansB* contributes to *S*. Typhi survival early in infection. Surprisingly, *ΔansB + ansB-F* did not fully recapitulate WT *S*. Typhi *in vivo* (Fig. 4E through G), despite its capacity to restore ISR activation in macrophages (Fig. 4A and B). Together, these findings establish a role for *S*. Typhi *ansB* in activating the ISR during murine macrophage infection, but also reveal that, at least for bacterial killing, deleting bacterial *ansB* was not equivalent to deleting host *Gcn2*. We therefore considered that another amino acid sensor might be engaged during *S*. Typhi infection, with the most likely candidate being the nutrient sensor mTOR.

### mTOR inhibition impairs ISR induction during *S*. Typhi infection

**Fig 5:**
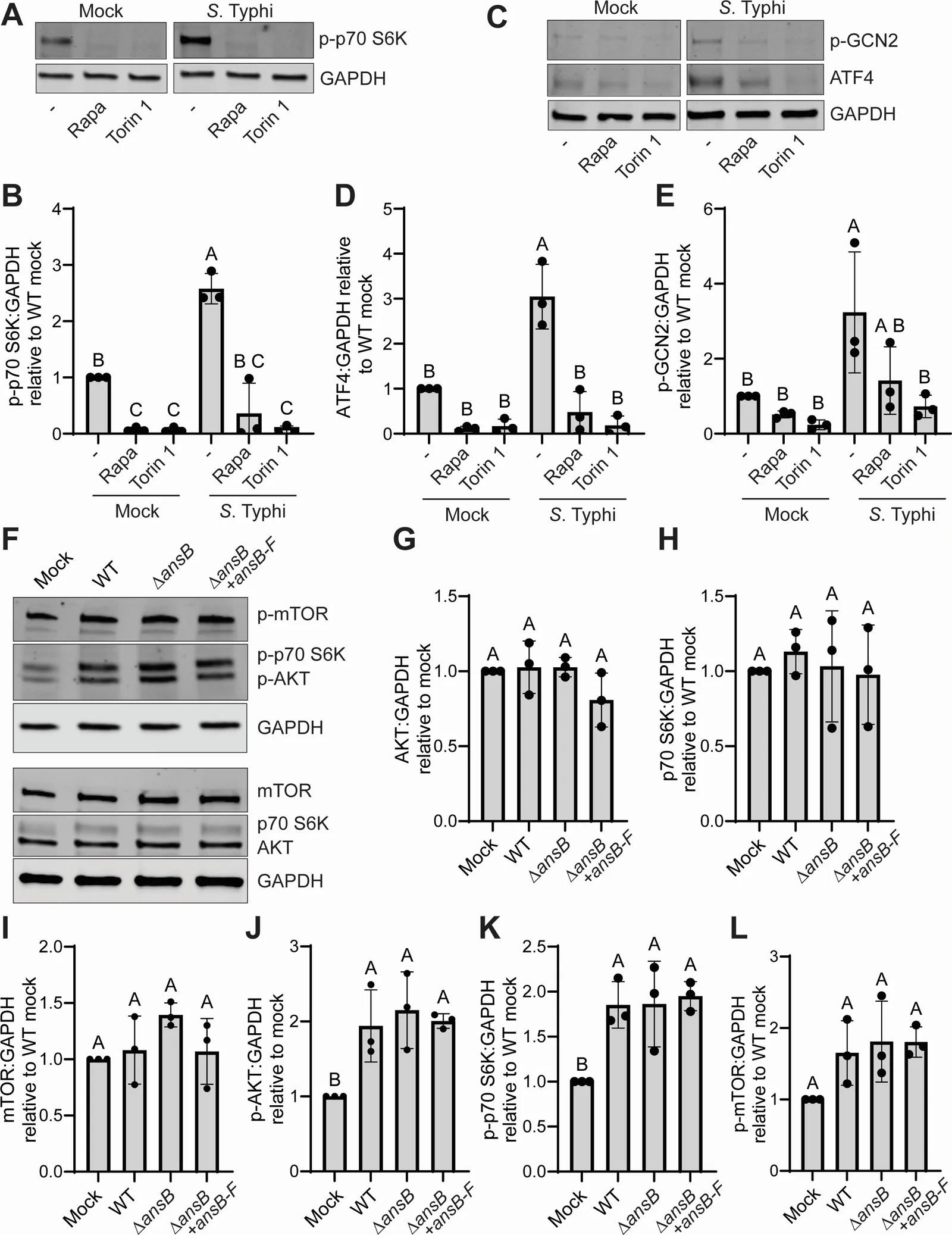
mTOR inhibition prevents *S*. Typhi-induced ISR activation in infected murine macrophages. (**A–E**) WT BMDMs were treated with mTOR inhibitors rapamycin (100 nM) or torin 1 (250 nM) added at the time of *S*. Typhi infection. Whole-cell lysates were harvested at 4 hpi followed by SDS-PAGE and immunoblot probed with the indicated antibodies. (**A**) Representative immunoblot of phospho-p70 S6K and (**B**) densitometry quantification of three experimental replicates. (**C**) Accompanying representative phospho-GCN2 and ATF4 immunoblots and (**D, E**) quantification of three experimental replicates of (**C**). (**F–L**) WT BMDMs infected with *S*. Typhi wild-type Ty2, *ΔansB*, or *ΔansB + ansB-F* at 4 hpi, followed by SDS-PAGE and immunoblotting with the indicated antibodies. (**F**) Representative immunoblot for mTOR, p70 S6K, AKT, and accompanying phosphorylated forms (mTOR Ser2448; p70 S6K Thr389; AKT Ser473). (**G–L**) Densitometry quantification of three experimental replicates of panel **F**. (**B, D, E, G–L**) Unpaired one-way ANOVA and Tukey’s post-test were used to compare column means with compact letter display for statistical representation of comparisons with *P*-value < 0.05 (51). For compact letter display, two groups marked with the same letter are not statistically different, whereas two groups marked with different letters are statistically different. Error bars represent SD.

**Fig 6:**
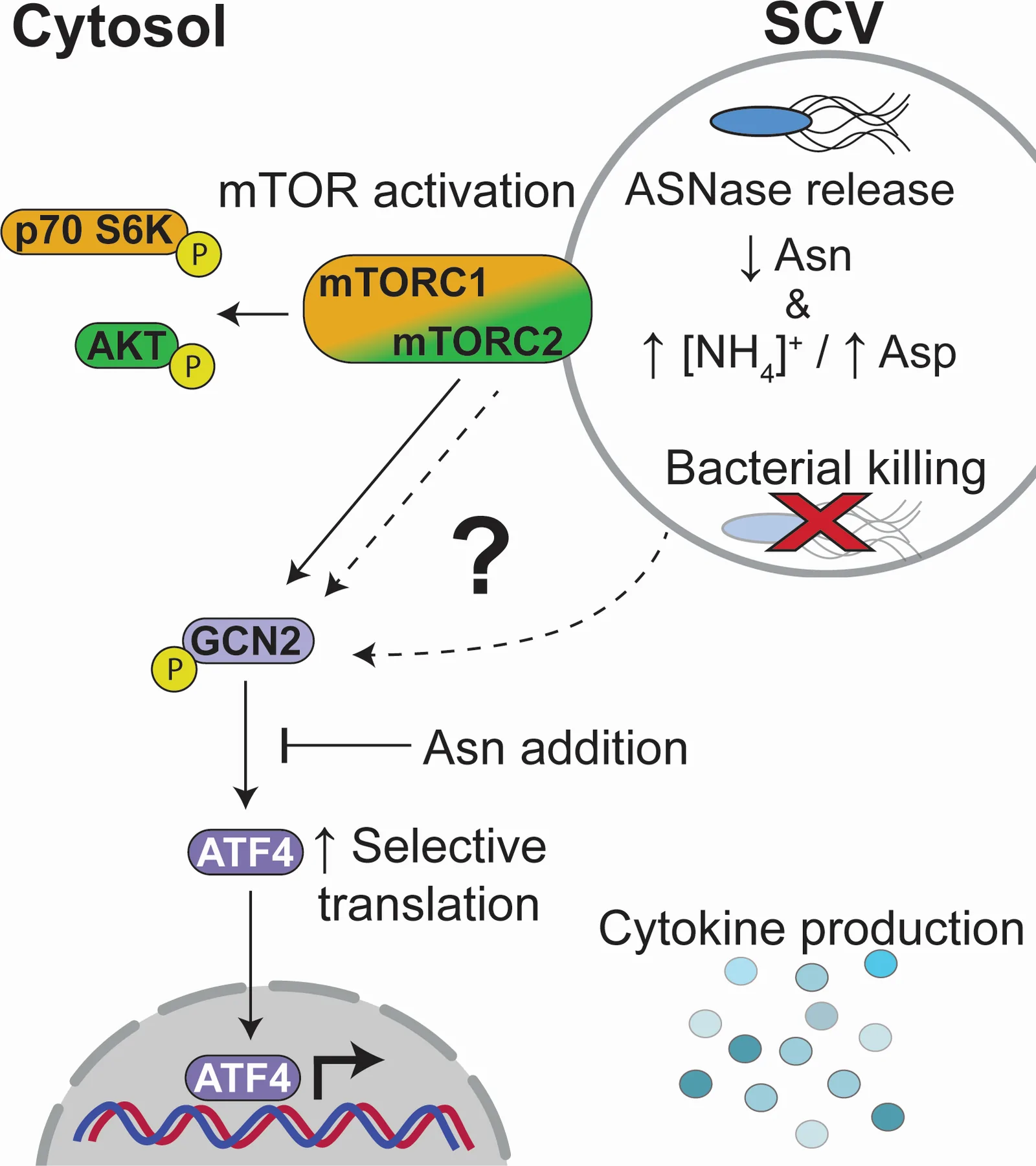
Model of *S*. Typhi ISR activation during murine macrophage infection. We propose the following non-exclusive models for *S*. Typhi induction of the ISR during murine macrophage infection. We hypothesized that the ISR is triggered by bacterial degradation of asparagine in the vacuole, leading to increased aspartic acid (Asp) and ammonia ([NH_4_]^+^) from *S*. Typhi AnsB. In the first model, mTOR at the SCV surface is activated by infection, and mTOR signaling is modified in an AnsB-dependent manner, e.g., changing bias from predominantly mTORC1 to mTORC2. The active mTOR complex directly or indirectly phosphorylates GCN2, activating the ISR. GCN2-specific programming promotes cytokine production, and murine macrophages lacking GCN2 have impaired *S*. Typhi restriction. In the second model, infection-induced mTOR licenses GCN2, while *S*. Typhi AnsB acts through an mTOR-independent pathway to induce GCN2 phosphorylation with the same effects on infection outcomes.

SCVs serve as a niche for *Salmonella* within infected macrophages (62). With 1–3 bacteria within the SCV of most infected cells at 8 hpi (Fig. S6), we questioned whether bacterial asparaginase could deplete cytosolic asparagine sufficiently to induce GCN2 and instead hypothesized that mTOR localized at the SCV membrane might sense asparaginase activity within the SCV. As GCN2 is a known substrate of mTOR, bacterial ASNase II activity might modulate signaling across the SCV membrane to mTOR to activate cytosolic GCN2 (63). To test this hypothesis, we treated *S*. Typhi-infected BMDMs with mTOR inhibitors and assayed p70 S6K phosphorylation (as a proxy for mTOR activation), GCN2 phosphorylation, and ATF4 induction. Treatment with the pan-mTOR inhibitor torin 1 completely ablated p70 S6K phosphorylation (Thr389) (Fig. 5A and B), GCN2 phosphorylation, and ATF4 induction (Fig. 5C through E), suggesting that mTOR is required in some way to sensitize GCN2 to Asn depletion. Treatment with the mTOR complex 1 (mTORC1)–selective inhibitor rapamycin also reduced ISR induction, but not below the mock-untreated baseline (Fig. 5C through E). Finally, we tested the capacity of *ΔansB S*. Typhi to increase mTOR activity by probing whole-cell lysates from infected BMDMs for p-mTOR, p-p70 S6K, and p-AKT (Ser473) (Fig. 5F through L). Lack of *ansB* did not alter mTOR phosphorylation compared to WT *S*. Typhi, suggesting that AnsB impacts GCN2 through mTOR complex composition or perhaps through an mTOR-independent mechanism (Fig. 5F through L). These results are consistent with a model in which mTOR is activated by *S*. Typhi infection of murine macrophages and is required for GCN2 induction, possibly through spatial regulation of mTOR complexes associated with the SCV. We hypothesize that *S*. Typhi AnsB depletion of asparagine either qualitatively modifies mTOR signaling to promote GCN2 activation or otherwise activates GCN2 that has been licensed by mTOR (Fig. 6).

## DISCUSSION

Amino acid sensing plays a critical role in cellular homeostasis and the innate immune response to infection. Here, we determine that live *S*. Typhi activates the ISR in a restrictive murine macrophage model of infection via the eIF2α kinase GCN2, dependent on mTOR activation. GCN2 was required to control bacterial infection and potentiate cytokine production early in macrophage infection. U-937 cells did not activate the ISR during *S*. Typhi infection but were capable of inducing the ISR under control conditions, highlighting GCN2 activation as one potential factor that distinguishes the murine vs human response. We found that GCN2 activation was suppressed or prevented by asparagine supplementation or deletion of the *S*. Typhi ASNase II *ansB*, respectively. GCN2-deficient mice remained as resistant to *S*. Typhi infection *in vivo* as wild-type controls. However, deletion of *S*. Typhi *ansB* reduced bacterial burden in the liver of infected animals. *S*. Typhi infection increased mTOR activity as measured by phosphorylation of substrate kinases p70 S6K and AKT, but deletion of *ansB* did not reduce mTOR, p70 S6K, or AKT phosphorylation, suggesting that mTOR may instead play a role in licensing or sensitizing GCN2 to ASNase activity. We therefore favor a model in which mTOR activation by *S*. Typhi is tuned by the products of bacterial ASNase activity to induce the ISR and promote macrophage inflammation and host defense (Fig. 6).

Bidirectional signaling between GCN2 and mTOR has previously been documented, but is poorly understood in the context of infection (31, 63–66). We initially proposed a linear hypothesis in which bacterial asparaginase activity depleted asparagine, leading to ISR activation. Instead, the results demonstrated a more complex signaling structure with mTOR upstream of ISR induction during *S*. Typhi infection of murine macrophages. Prior studies have established mTOR activation during *S*. Typhimurium infection of human macrophages and epithelial cells, as well as murine macrophages (38, 67–70), including recruitment of mTOR to the SCV through secretion of effectors such as SseJ and SopB (71, 72) or activation of host focal adhesion kinase (68). While *S*. Typhi activates mTOR in human THP-1 cells, this has not previously been reported in murine macrophages (73). Multiple Type III effector proteins secreted by *S*. Typhimurium are pseudogenes in *S*. Typhi, including *sseJ*, opening up the possibility for serovar-specific mTOR modulation (74, 75). We therefore consider two non-exclusive models in which SCV-associated mTOR may directly or indirectly activate GCN2 by sensing AnsB activity through buildup of degradation products, aspartate and/or ammonia, within the SCV lumen (29, 76). First, *S*. Typhi AnsB activity may determine the functional composition of the mTOR complex, perhaps preferentially activating mTORC2 over mTORC1. Coupled with previous findings that recombinant ASNases impair mTORC1 in some cancer cells (77, 78), we favor a model where mTORC2 signaling at the SCV is primarily responsible for the observed ISR activation during *S*. Typhi infection. Despite evidence of mTORC2 spatially localizing to a subpopulation of vesicles, lack of an mTORC2-specific inhibitor has impeded mechanistic disentanglement of mTORC2 signaling from the ISR and mTORC1 activity (30, 79). Secondly, it is possible that *S*. Typhi infection activates mTOR irrespective of AnsB activity and licenses GCN2 in some way, while AnsB then acts through an mTOR-independent pathway to signal GCN2 activation. It may be that activating GCN2 artificially with halofuginone in human U-937 macrophages did not successfully mimic the coordinated regulation that occurs during infection in murine macrophages, and therefore did not trigger the anti-microbial function observed in murine macrophages. Our two proposed models are consistent with observations that, during *S*. Typhi infection, both AnsB and mTOR activity are required for phosphorylation of GCN2 and induction of the ISR.

Spatial localization of signaling enables cells to distinguish between extracellular and intracellular environmental cues. Internalization of plasma membrane receptors via phagocytosis allows for combinatorial signaling, as machinery of the endolysosomal sensor network, such as mTOR, is recruited to the phagosome. Intriguing work has demonstrated that *S*. Typhimurium ASNase II depletion of L-asparagine during co-culture with T cells is necessary and sufficient to impair T cell function (58, 60, 61). However, T cells do not support *Salmonella* invasion, and both WT and *ΔansB* bacteria invade poorly during T cell co-culture (60). In our macrophage model of *S*. Typhi intracellular infection, we find that *S*. Typhi may also shape the innate immune response via ASNase II activity, potentially using a distinct signaling network. Our data are consistent with a model in which a combination of innate immune sensing, coupled with the signature of ASNase II activity, acts as an alarm for the host cell to identify a state of inflammatory nutrient stress indicative of bacterial infection.

As GCN2 and mTOR both control metabolic reprogramming, it is likely that modulation of these pathways by *S*. Typhi shapes macrophage immunometabolic responses. GCN2 and the ISR exert metabolic control upon activation through transcriptional and translational regulation, which cooperate to overcome cellular stress (21). In addition to lipid metabolism and glucose homeostasis, a prominent target of ISR regulation is asparagine synthetase (ASNS) (21, 80). The idea of ASNS regulation by the ISR is intriguing, given our finding that *S*. Typhi ASNase II is required for ISR induction, suggesting that macrophages may benefit from sensing depleted asparagine to promote host defense but then must replenish their pools. mTOR also controls many macrophage functions through metabolic and cell cycle regulation (81). Macrophages lacking mTORC1 increase inflammatory cytokine production in response to LPS, despite lower glycolytic activity, whereas mTORC2 is required for polarization of alternatively activated M2 macrophages *in vivo* (82, 83). Additionally, when mTOR is inactivated, autophagy is induced to degrade intracellular components, such as organelles, damaged proteins, and vesicular cargo, to conserve energy and recycle resources (84). Bacterial pathogens such as *Salmonella* may be eliminated through autophagic activity, and multiple *Salmonella* effectors reinforce mTOR activation to escape autophagy-mediated killing (11, 71, 72). While our data support a GCN2-dependent model for *S*. Typhi restriction downstream of mTOR activation, our model does not rule out bidirectional signaling between GCN2 and mTOR to inactivate SCV-associated mTOR, promoting *Salmonella* killing through autophagy (64, 65). Future studies will elucidate the complex nutrient sensing mechanisms that coordinate the murine macrophage response to *S*. Typhi infection and determine how engagement of this pathogen with human macrophages may shape the immune environment to be more permissive to chronic infection.

## MATERIALS AND METHODS

### Reagents and consumables

Detailed information for antibodies, reagents, and consumables used in this study is found in Tables 1 and 2.

**TABLE 1:** Antibodies^*a*^

Target	Catalog number	Manufacturer	Concentration
ATF4 (D4B8)	11815	Cell Signaling	WB: 1:1,000; IF: 1:200
p-GCN2 (Thr899) (EPR2320Y)	ab75836	Abcam	WB: 1:1,000
GAPDH (6C5)	sc-32233	Santa Cruz	WB: 1:5,000
FLAG (M2)	F1804	Sigma-Aldrich	WB: 1:1,000
p70 S6K	9202	Cell Signaling	WB: 1:1,000
p-p70 S6K (Thr389) (108D2)	9234	Cell Signaling	WB: 1:1,000
AKT (pan) (40D4)	2920	Cell Signaling	WB: 1:1,000
p-AKT (Ser473) (193H12)	4058	Cell Signaling	WB: 1:1,000
mTOR (7C10)	2983	Cell Signaling	WB: 1:1,000
p-mTOR (Ser2448) (D9C2)	5536	Cell Signaling	WB: 1:1,000
Goat anti-rabbit IRDye 680RD	926-68071	LI-COR	WB: 1:5,000
Goat anti-mouse IRDye 800CW	926-32210	LI-COR	WB: 1:5,000
Goat anti-rabbit IgG (H + L) cross-adsorbed secondary antibody, Alexa Fluor 647	A21244	Invitrogen	IF: 1:2,000

^*a*^ WB, western blot. IF, immunofluorescence.

**TABLE 2:** Reagents and consumables

Product name	Catalog number	Supplier
Sodium pyruvate	11360070	Invitrogen
Dulbecco’s Modified Eagle Medium (DMEM)	12430054	Invitrogen
2-Mercaptoethanol	21985023	Invitrogen
Penicillin/streptomycin (pen/strep)	15140122	Invitrogen
RPMI 1640	11875119	Invitrogen
Gentamicin	15750060	Gibco
Tunicamycin	11445	Cayman Chemical
Tween-20	BP337-100	Fisher Scientific
TRIS-buffered saline (TBS)	J60877.K3	Thermo Scientific
Bovine serum albumin (BSA)	BP1600-100	Fisher Scientific
Nitrocellulose	10600002	Amersham
Extra thick blotting paper	1703965	Bio-Rad
4–20% Mini-PROTEAN TGX protein gels	4561093, 4561096	Bio-Rad
Dulbecco’s phosphate-buffered saline with calcium chloride and magnesium chloride ions (PBS+/+)	14040133	Invitrogen
Dulbecco’s phosphate-buffered saline, no calcium, no magnesium (PBS−/−)	14190250	Invitrogen
Dimethyl sulfoxide (DMSO)	D2650-100	Sigma-Aldrich
#1.5 square cover glass	12541013	Fisher Scientific
16% paraformaldehyde	15710	Electron Microscopy Sciences
Triton X-100	T9284	Sigma-Aldrich
Phalloidin-iFluor 488 Reagent	ab176753	Abcam
4′,6-Diamidino-2-phenylindole (DAPI)	62248	Thermo Scientific
Fisherbrand Superfrost Plus Microscope Slides	12-550-15	Fisher Scientific
ProLong Glass Antifade Mountant	P36980	Invitrogen
PhenoPlate 96-well	6055302	Revvity
6-well cell culture-treated plates	FB012927	Fisher Scientific
24-well cell culture-treated plates	FB012929	Fisher Scientific
Phorbol 12-myristate 13-acetate (PMA)	P1585	Sigma-Aldrich
Halofuginone	5.05763	Sigma-Aldrich
MILLIPLEX Mouse Cytokine/Chemokine Magnetic Bead Panel	MCYTMAG70PMX25BK	Milliplex
4× Laemmli Sample Buffer	1610747	Bio-Rad
Prec Plus Protein Kaleidoscope Stds	1610375	Bio-Rad
Normal goat serum	PCN5000	Invitrogen
CB 300 Lithium Heparin Microvette	16.443.100	Thermo Scientific
1.0 mm zirconia/silica beads	1107911z	BioSpec Products
Screw-Top Tubes with O-Ring Cap	HS10060	Millipore Sigma
Minimum Essential Medium (MEM)	11095080	Invitrogen
BamHI-HF restriction enzyme	R3136S	New England Biolabs
NotI-HF restriction enzyme	R3189S	New England Biolabs
Herculase II Fusion DNA Polymerase	600679	Invitrogen
T5 exonuclease	M0363S	New England Biolabs
Phusion polymerase	M0530S	New England Biolabs
Taq DNA ligase	M0208L	New England Biolabs

### Bacterial culture for infections

Detailed information for bacterial strains used in this study is found in Table 3. Bacterial stocks were stored at −80°C in Luria Broth (LB) + 20% glycerol and plated on LB + agar for use with 100 µg/mL ampicillin as needed. A single bacterial colony was inoculated into 3 mL of LB with 100 µg/mL ampicillin as needed and grown overnight, slanted, shaking, at 37°C. The next day, 1 mL of culture was pelleted, the supernatant removed, and the bacteria were resuspended to known CFU/OD_600_ in PBS+/+. Each experimental use of bacteria was accompanied by CFU plating and overnight incubation to confirm infectious dose. Heat-killed bacteria were prepared by resuspending in PBS followed by incubation at 70°C for 1 h, and killing was confirmed by plating on LB agar with overnight incubation.

**TABLE 3:** Bacterial strains

Strain	Alternative names; description
WT *Salmonella* Typhi (Ty2)	MOR719; parental strain for *ansB* and *ansP* mutants
WT *Salmonella* Typhimurium (strain SL1344)	MOR23, Portnoy strain #4476;
*S*. Typhi (Ty2) *ansB* clean deletion	MOR720, JS0171;
*S*. Typhi (Ty2) *ansB* T111A	MOR722, JS0173; *ansB* catalytic dead point mutant
JS0171 *ansB-F*	MOR723, JS0174; *ansB* clean deletion and in-locus complementation with *ansB*-FLAG.
*S*. Typhi (Ty2) *ansP*	MOR724, JS0175; *ansP* clean deletion
*S*. Typhi (Ty2) pGEN222::Pem7-DsRed	MOR603; constitutive fluorescent DsRed (85) expression. Ampicillin resistant.
*S*. Typhimurium (SL1344) pGEN222::Pem7-DsRed	MOR503; constitutive fluorescent DsRed (85) expression. Ampicillin resistant.

### Bacterial cloning

Bacterial mutants were generated as previously described (86–88). Briefly, pSB890 (Tet^R^ and *sacB* for sucrose selection) served as the backbone for gene deletions, point mutations, and FLAG tag insertions. The vector was linearized using restriction enzymes (BamHI-HF and NotI-HF). Inserts for each mutant were amplified by PCR using Herculase II Fusion DNA Polymerase and specific primers with the *S*. Typhi Ty2 genome as the template. The digested vector and inserts were then assembled using Gibson assembly mix (T5 exonuclease; Phusion polymerase; Taq DNA ligase). The resulting plasmids were transformed into *E. coli* ß2163 ∆nic35 for conjugation, followed by subsequent homologous recombination in *S*. Typhi Ty2. All strains were verified by Sanger sequencing through the Cornell Institute Biotechnology Resource Center (BRC) Genomics Facility (RRID: SCR_021727). The locus tag names for *ansB* and *ansP* in the genome of *S*. Typhi Ty2 are T_RS15300 (formerly t3018 or STY3259 in the strain CT18) and T_RS07610 (also known as t1494 or STY1481 in the strain CT18), respectively. The amino acid sequences of AnsB and AnsP can be found at https://www.uniprot.org/uniprotkb/Q8XGY3/entry and https://www.uniprot.org/uniprotkb/Q8Z739/entry (86).

### Media formulations

Bone marrow media (BMM) was prepared with 20% heat-inactivated FBS, 30% L-929 conditioned media, 1% sodium pyruvate, 0.1% 2-mercaptoethanol, and the remainder DMEM containing high glucose, L-glutamine, HEPES, and phenol red. BMM may sometimes be supplemented with 1% penicillin/streptomycin (pen/strep) as noted. L-929 cells were cultured in MEM supplemented with 1% NEAA, 1%, 2 mM L-glutamine, 10 mM HEPES, and 10% FBS. L929-conditioned medium was sterile filtered prior to use.

D10 medium was prepared with 10% heat-inactivated FBS, 1% sodium pyruvate, 0.1% 2-mercaptoethanol, and the remainder DMEM containing high glucose, L-glutamine, HEPES, and phenol red. Similarly, U-937 complete medium was prepared with 10% heat-inactivated FBS, 0.1% 2-mercaptoethanol, and the remainder RPMI 1640.

### Bone marrow isolation and macrophage differentiation

Femurs and tibias from male and female mice aged 8–12 weeks were isolated, and bone marrow was extracted by flushing with PBS+/+ with pen/strep. Cells were centrifuged at 250 × *g* for 5 min at 4°C and resuspended in BMM media with pen/strep. Cells were divided into 15 cm non-tissue culture treated dishes at a density of ~3 × 10^6^ nucleated cells/dish in 25 mL BMM + pen/strep. BMM is made with L-929-conditioned media, which contains monocytic colony-stimulating factor to promote macrophage differentiation (89, 90). For *Gcn2^−/^*^−^ bone marrow isolation, 1% NEAA was added to BMM (91). Monocytes were differentiated for a total of 6 days, with 30 mL of additional BMM with pen/strep added on day 3. On day 6, cells were washed twice with cold PBS−/−, then 10 mL PBS−/− was added, and plates were chilled at 4°C for 5 min. Cells were then lifted by washing and pelleted at 250 × *g* for 10 min, resuspended in BMM with 10% FBS and 10% DMSO, and frozen in 1.5 × 10^7^ cell/mL aliquots in cryogenic vials. Aliquots were frozen in −80°C for 24 h in a Mr. Frosty freezing container before transferring to liquid nitrogen for long-term storage.

### Bone marrow-derived macrophage cell culture and infections

Cells were thawed in a 37°C water bath and diluted 1:10 in prewarmed D10. Cells were pelleted at 250 × *g* for 5 min, counted, plated at 5 × 10^5^ cells/mL in D10 media in the appropriate cell culture plate (noted per protocol), and incubated overnight at 37°C, 5% CO_2_, with humidity. The following day, the overnight medium was removed and replaced with an equal volume appropriate for the vessel. Where used, bacteria or heat-killed equivalents were delivered at MOI 10. Where used as a control, tunicamycin was dosed at 5 µM. Where used, NEAAs and EAAs were added at 1× final concentration using commercial stocks (Table S1). Where supplemented as individual amino acids, stocks were made to match the concentrations of the commercial products found in Table S1 and used at 1× final concentration. Following treatment addition, infection was synchronized by centrifugation at 500 × *g* for 5 min. After 30 min of incubation, cells were washed twice with PBS+/+, and D10 with gentamicin (50 µg/mL) was added. Plates were incubated for an additional 30 min, washed twice with PBS+/+, and D10 with gentamicin (5 µg/mL) was added. Cells were returned to incubate for the remainder of the experiment as indicated. At the time of harvest, cells were washed twice with PBS+/+.

### U-937 cell culture and infections

#### Adaptation of U-937 cells to D10 media

The U-937 cell line used in this study was acquired from ATCC (catalog number CRL-1593.2) circa 2007 and validated using the ATCC Cell Line Authentication Service immediately prior to the submission of this study. U-937 monocytes were acclimated from ATCC-recommended complete medium to D10. The U-937 medium recipe contains RPMI 1640, which has nearly three times the concentration of L-asparagine demonstrated herein to suppress ISR induction (0.378 mM vs 0.100 mM, Tables S1 and S2), making the switch to D10 necessary. Previous studies have demonstrated the capacity for THP-1 cells (another human monocytic cell line) to adapt to DMEM-based media (92). Every second passage, the percentage of D10 to ATCC complete medium was increased by 25%. Doubling time, live cell diameter, and cell viability were measured to ensure cellular health (data not shown). Once 100% D10 was achieved, doubling time recovered to ~45 h within five passages, and cells were determined to be suitable for use. U-937 cells were maintained between 2.0 × 10^5^ and 1.0 × 10^6^ cell/mL, and experimental replicates were performed within five passages.

#### Infection of U-937 cells

D10-acclimated U-937 cells were plated at 5 × 10^5^ cells/mL in 2 mL D10 media in a 6-well cell culture plate with PMA (100 nM) and incubated for 24 h at 37°C, 5% CO_2_, with humidity. PMA was removed, and cells were washed twice with PBS+/+, then 2 mL D10 was replaced, and cells were allowed to recover for 24 h. The following day, U-937 cells were infected using the same method as BMDMs. Where indicated, 80 nM halofuginone was added for the first 2 h of infection before withdrawal by washing twice with PBS+/+ and replacing with D10.

### Fluorescence microscopy

For Fig. 1C and D, cells were plated in 6-well plates at 5 × 10^5^ cells/mL in 2 mL per well containing glass coverslips and incubated overnight. Following 8 h of infection, cells were fixed for 15 min in freshly prepared 4% paraformaldehyde and permeabilized with PBS containing 0.1% Triton X-100 (wash buffer). Coverslips were blocked with wash buffer containing 3% BSA and 5% normal goat serum (block buffer). Primary ATF4 antibody was incubated overnight at 4°C in block buffer. Coverslips were washed, and secondary antibody, phalloidin (1:5,000), and DAPI (1:1,000) in block buffer were incubated at room temperature for 1 h in the dark. Coverslips were washed and mounted on glass slides using Prolong Glass, then cured at room temperature in the dark for 24 h before storage at 4°C. Confocal microscopy was performed using a Nikon X1 Yokogawa spinning disk microscope at 60× magnification.

For all other figures, fluorescence microscopy was performed as above with the following alterations: cells were plated in 96-well PhenoPlate at 5 × 10^5^ cells/mL in 0.1 mL per well. After washing away secondary staining, wells were stored in 100 µL water until imaging. The water was refreshed before imaging on the Yokogawa CQ1 automated high-content spinning disk confocal microscope at 40× magnification. For each image, a 10-slice Z-stack covering the cell height was acquired, and a maximum intensity projection was created for analysis, with 25–35 fields per well. These conditions resulted in more than >1,000 cells imaged per condition per experimental replicate.

### Image analysis

Representative microscopy images prepared using FIJI software with automated macros developed for this study and may be found at https://github.com/zmpowers/software-for-GCN2. Briefly, input image pixel intensities are set to user-determined values, and the representative area of the image is chosen by the user to crop. The cropped selection is saved as single channel .tif and false colored .png files, along with a composite false colored .png of non-ATF4 channels (DAPI, phalloidin, DsRed *S*. Typhi). This process is repeated for all representative images in the experiment to keep pixel values across images consistent.

Image analysis was performed with CellProfiler v4.2.8 using analysis pipelines documented here: https://github.com/zmpowers/software-for-GCN2 (93). Briefly, nuclei were identified by DAPI, and the cytosol was identified by phalloidin with nuclear masking. Objects touching the image boundary were discarded from analysis. Nuclear ATF4 signal was measured. ROUT analysis was performed on ATF4 nuclear signal with *Q* = 1% and outliers excluded. To calculate the percentage of ATF4-positive nuclei, a threshold was calculated using the 95th percentile intensity of the mock-treated condition per experimental replicate, thereby making 95% of mock-treated cell nuclei as ATF4-negative. This threshold was propagated to the experimental conditions, and the proportion of nuclei with intensities above this threshold is represented as “% ATF4-positive nuclei.” Points represent the mean % ATF4-positive nuclei per experimental replicate.

For Fig. 1D, the nucleus and cytosol were identified as described above. The mean ATF4 signal was measured within the nuclei and cytosol, and a nuclear:cytosolic ratio was calculated. The mean nuclear:cytosolic ATF4 signal from the mock-treated condition was calculated and used as a threshold. To determine the percentage of ATF4-positive nuclei, this threshold was applied against the nuclear:cytosolic ATF4 signal for each cell and is displayed as a proportion of positive cells analyzed from that condition.

### Bacterial killing and cytokine analysis

Cells were plated in 24-well cell culture plates with 5 × 10^5^ cells/mL in 0.5 mL per well. Cell culture and infection were performed using BMM in place of D10 for killing assays and cytokine collection. At 1 h and 24 h post-infection, supernatants were collected for ELISA analysis, and cells were washed twice using PBS+/+ before cells lysed in 500 µL PBS containing 1.0% Triton X-100. A 10-fold dilution series was performed, lysates were plated on LB + agar, and incubated overnight at 37°C before CFU counting. Enzyme-linked immunosorbent assays (ELISAs) and Luminex cytokine analysis were performed by the UMICH Immune Monitoring Shared Resource core facility.

### Immunoblotting and quantification

Cells were plated in 6-well plates with 5 × 10^5^ cells/mL in 2 mL per well. Following infection, whole-cell lysates were collected in 1× Laemmli buffer and stored at −80°C. Before use, samples were heated to 95°C for 5 min. Samples were run on 4–20% Mini-PROTEAN TGX protein gels in TRIS-glycine-SDS buffer for 45 min at 100 V. Proteins were transferred to nitrocellulose membranes using the Bio-Rad Trans-Blot Turbo system (25 V limit, 2.5 A constant, 20 min). Membranes were blocked for 30 min at room temperature in 5% BSA in 1× TBS. Primary antibodies were incubated overnight at 4°C in TBS with 5% BSA using antibodies found in Table 1. Following incubation, membranes were washed with TBS with 0.1% Tween-20 (TBS-Tw). Secondary antibodies were added in TBS-Tw with 5% BSA and incubated for 1 h at room temperature. Blots were washed with TBS-Tw before imaging (LI-COR Odyssey Classic, medium quality, 84 μm). Quantification was performed using FIJI image analysis software (94). Briefly, the mock lane was selected using the rectangle tool and a box of the same size was propagated to all lanes. Peaks of interest were chosen by drawing a line at the inflection point on either side of the peak base, and the area under the curve was measured using the wand tool. A (protein of interest:GAPDH) ratio was calculated, and experimental conditions were normalized to mock.

### Murine strains

C57BL/6J (strain #000664, WT) and B.6129S6-Eif2ak4^tm1.2Dron^/J (strain #008240, *Gcn2^−/^*^−^) mice were purchased from Jackson Laboratory. Mice with mutations in both *Pkr* and *Gcn2* (“double-knockout mice”) were produced as follows: B6/J;C57BL/6NCrl-Eif2ak2^em1(IMPC)Mbp/Mmucd^ mice (PKR-TKO mice) (95) were crossed to B6.129S6-Eif2ak4^tm1.2Dron^/J. The genotypes of parental mice during the breeding process were determined from tail DNAs (Transnetyx). The resulting double-knockout mice are designated PKR-TKOxGCN2*^−/^*^−^ (*Pkr^−/−^Gcn2^−/^*^−^). Their genotypes were confirmed by analysis of tail DNA by Transnetyx. Immunoblotting of bone marrow-derived macrophages from the PKR-TKOxGCN2*^−/^*^−^ mice confirmed that they did not express detectable PKR or GCN2 (data not shown). Genotyping PCR was performed using the primers for *Gcn2* and *Pkr* found in Table 4.

**TABLE 4:** Oligonucleotides

Name	Sequence	Description	Source
dansB_F1	TAAAAAGCCCCACCGCGGTGGCGGCCCTGCGCCAGCAATCCATTGTCGAACC	*ansB deletion*	This study
dansB_R1	CCAGTTGACATAACTGGAGATATAACAGATAATGCCCCGGTCGGAAGGCCG	*ansB* deletion	This study
dansB_F2	GTTATATCTCCAGTTATGTCAACTGGTCGC	*ansB* deletion	This study
dansB_R2	GTAAGTGAACTGCAGCCCGGGGGATCCGTATCTGCGCCTTTTTATTCTGTTTTTTCC	*ansB* deletion	This study
ansB_T111A_F2	CTCTTCCATCGTATCAGCACCGTGGGTGATCACG	*ansB* point mutation	This study
ansB_T111A_R1	CGTGATCACCCACGGTGCTGATACGATGGAAGAG	*ansB* point mutation	This study
ansB-Flag_F2	TTACTTGTCGTCATCGTCTTTGTAGTCCATATACTGATTGAACATCGTCTGGATCTG	*ansB-F* allele	This study
ansB-Flag_R1	ATGGACTACAAAGACGATGACGACAAGTAAAGATAATGCCCCGGTCGGAAGGCCG	*ansB-F* allele	This study
dansP_F1	TAAAAAGCCCCACCGCGGTGGCGGCCCAAATTTTGCTCAATAGCTTTATATGTCCTAC	*ansP* deletion	This study
dansP_R1	TACGTTACCTGTTATAGCCTGTCCTGAC	*ansP* deletion	This study
dansP_F2	GGACAGGCTATAACAGGTAACGTACATTACCCTTCCCCGGCAGCCTCAG	*ansP* deletion	This study
dansP_R2	GTAAGTGAACTGCAGCCCGGGGGATCATCGACACGTGAACAAGAAGCGACGC	*ansP* deletion	This study
CR_Eif2ak2_comF	GAGGTCCTGAGCACATTGTCAGTG	*Pkr* Forward	(96)
CR_Eif2ak2_wtR*	CGTCCATCTAATGCACACTTTGAAAAG	*Pkr* Reverse	(96)
CR_Eif2ak2_mutR	CCATCACTGTTCACAGGGAGCTAAC	*Pkr* Mutant Reverse	(96)
oIMR8791	TGCCACTGTCAGAATCTGAAGCAGG	*Gcn2* Mutant Reverse	(97)
oIMR8796	TCTCCCAGCGGAATCCGCACATCG	*Gcn2* Forward	(97)
oIMR8797	ATCCAGGCGTTGTAGTAGCGCACA	*Gcn2* Reverse	(97)

### Murine infections

Bacteria were cultured overnight at 37°C with shaking in 3 mL LB with 10 mM NaCl to induce capsule expression. The following day, 1 mL of pelleted culture was washed twice with PBS+/+ and diluted to the calculated density, as measured by OD_600_ spectrophotometry. Mice were injected intraperitoneally with *S*. Typhi in 200 µL PBS+/+. Inoculums are as follows: Fig. 4E through G, 5 × 10^6^CFU/mouse; Fig. S1A-K, 4 × 10^7^ CFU/mouse. After 24 h of infection, mice were sacrificed, blood was collected in lithium heparin cuvettes via cardiac puncture, and plasma was isolated for cytokine analysis after separation by centrifugation at 8,000 × *g* for 5 min. Organs were homogenized in tissue disruption tubes with 1.0 mm zirconia/silica beads using the Omni Bead Ruptor 24 (*S* = 6.00; *C* = 01; *T* = 0:00; *D* = 0:00), and plated on LB agar. CFUs were counted following overnight incubation at 37°C. Outliers were analyzed by Robust Regression and Outlier Removal (ROUT) analysis with *Q* = 1% and were excluded.

### Statistical analysis and graphing

Statistical analysis calculations were performed in GraphPad Prism v10.4.1 (627) for Windows (GraphPad Software, Boston, Massachusetts, USA; www.graphpad.com). Where used, compact letter display comparisons note statistically significant differences (*P* < 0.05). Compact letter display statistical representation signifies differences by assigning letters between statistically different groups (51). If a letter is shared by two or more groups, the comparison is not statistically significant. If groups have no shared letters, the comparison is considered significant at the tested *P*-value (<0.05).

## Data Availability

The U-937 cell line used in this study was acquired from ATCC (catalog number CRL-1593.2) circa 2007 and validated using the ATCC Cell Line Authentication Service immediately prior to the submission of this study. The data sets presented and bacterial strains used in this study are available from the corresponding author upon request. Code generated for, and used within, this study for image analysis and figure preparation is made available on GitHub: https://github.com/zmpowers/software-for-GCN2.

## SUPPLEMENTAL MATERIAL

Supplemental material iai.00178-26-s0001.pdf Fig. S1 to S6; Tables S1 and S2.

ASM does not own the copyrights to Supplemental Material that may be linked to, or accessed through, an article. The authors have granted ASM a non-exclusive, world-wide license to publish the Supplemental Material files. Please contact the corresponding author directly for reuse.
