# Supplementary material for: Salmonella Typhi asparaginase-dependent activation of GCN2 promotes bacterial killing in murine macrophages
Source: Infect Immun. 2026 Jun 15;94(7):e00178-26. doi: 10.1128/iai.00178-26 (PMC13367068; doi:10.1128/iai.00178-26)
Supplement: Supplemental material — Fig. S1 to S6; Tables S1 and S2. [file iai.00178-26-s0001.pdf]

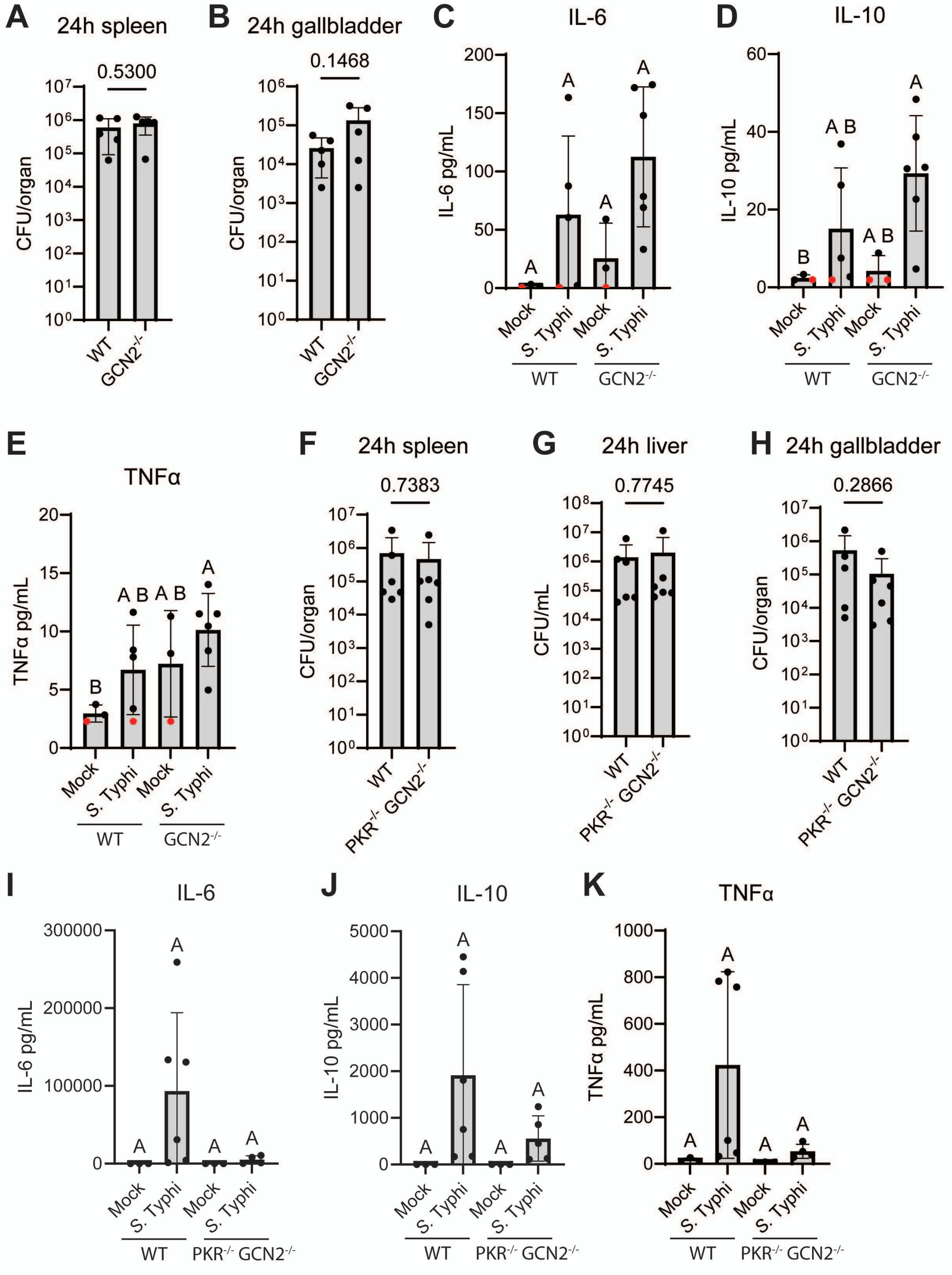
A
24h spleen
0.5300
CFU/organ
WT
GCN2-/-
B
24h gallbladder
0.1468
CFU/organ
WT
GCN2-/-
C
IL-6
IL-6 pg/mL
Mock
S. Typhi
Mock
S. Typhi
WT
GCN2-/-
D
IL-10
IL-10 pg/mL
Mock
S. Typhi
Mock
S. Typhi
WT
GCN2-/-
E
TNFα
TNFα pg/mL
Mock
S. Typhi
Mock
S. Typhi
WT
GCN2-/-
F
24h spleen
0.7383
CFU/organ
WT
PKR-/- GCN2-/-
G
24h liver
0.7745
CFU/mL
WT
PKR-/- GCN2-/-
H
24h gallbladder
0.2866
CFU/organ
WT
PKR-/- GCN2-/-
I
IL-6
IL-6 pg/mL
Mock
S. Typhi
Mock
S. Typhi
WT
PKR-/- GCN2-/-
J
IL-10
IL-10 pg/mL
Mock
S. Typhi
Mock
S. Typhi
WT
PKR-/- GCN2-/-
K
TNFα
TNFα pg/mL
Mock
S. Typhi
Mock
S. Typhi
WT
PKR-/- GCN2-/-

**Supplemental Figure S1: Bacterial burden and cytokine levels from *Gcn2*$^{-/-}$ and *Pkr*$^{-/-}$ *Gcn2*$^{-/-}$ *in vivo S.* Typhi infections. (A-E)** WT and *Gcn2*$^{-/-}$ mice infected with wildtype *S.* Typhi 4x$10^7$ CFU/animal by intraperitoneal injection. After 24h, animals were euthanized and homogenates of spleen **(A)** and gallbladder **(B)** were plated for CFUs. Serum was collected and analyzed by Luminex; values for IL-6 **(C)**, IL-10 **(D)**, and TNFα **(E)** are shown. **(F-K)** WT and *Pkr*$^{-/-}$ *Gcn2*$^{-/-}$ double knockout mice were infected with 4x$10^7$ CFU/animal of wildtype *S.* Typhi by intraperitoneal injection. After 24h, animals were euthanized and homogenates of spleen **(F)**, liver **(G)**, and gallbladder **(H)** were plated for CFUs to determine bacterial burden. **(I-K)** Serum from infected mice was analyzed by ELISA for IL-6 **(I)**, IL-10 **(J)**, and TNFα **(K)**. Red points indicate samples at or below the limit of detection. Unpaired one-way ANOVA and Tukey's post-test comparing column means with compact letter display statistical representation of comparisons with p-value < 0.05 (95). Error bars represent SD.

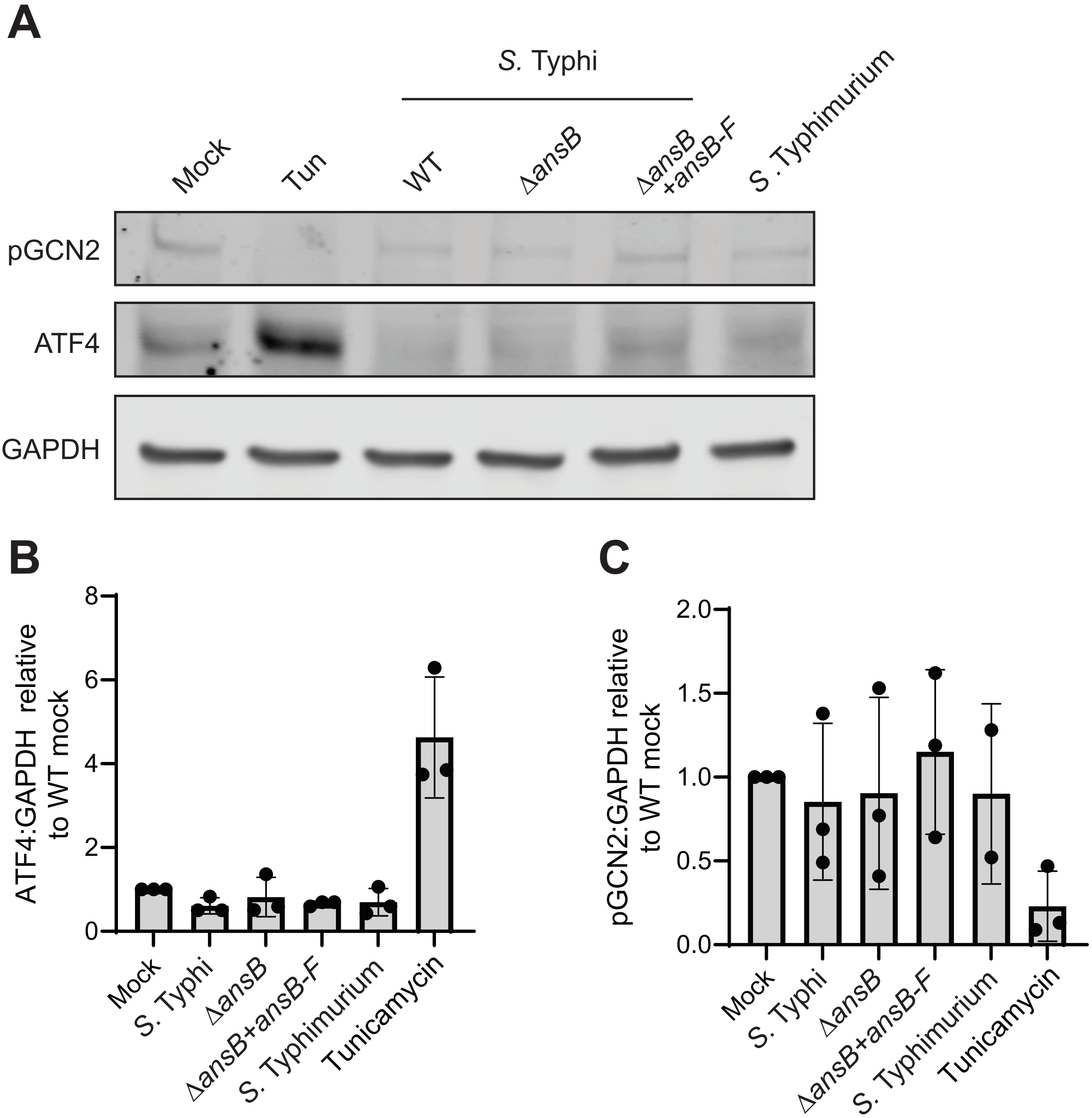
A
S. Typhi
Mock
Tun
WT
ΔansB
ΔansB +ansB-F
S. Typhimurium
pGCN2
ATF4
GAPDH
B
ATF4:GAPDH relative to WT mock
0
2
4
6
8
Mock
S. Typhi
ΔansB
ΔansB+ansB-F
S. Typhimurium
Tunicamycin
C
pGCN2:GAPDH relative to WT mock
0.0
0.5
1.0
1.5
2.0
Mock
S. Typhi
ΔansB
ΔansB+ansB-F
S. Typhimurium
Tunicamycin

**Supplemental Figure S2: U-937 human monocytic cells do not induce the ISR during *Salmonella* infection.** PMA-differentiated U-937 cells acclimated to D10 media were treated with the indicated *Salmonella* strains at MOI 10 or tunicamycin for 8h. **(A)** Cells were lysed and analyzed by SDS-PAGE and immunoblot with the indicated antibodies. Representative immunoblot for ATF4, phospho-GCN2, and loading control GAPDH is shown. **(B)** Densitometry quantification of ATF4 and **(C)** phospho-GCN2 from 3 experimental replicates of (A).

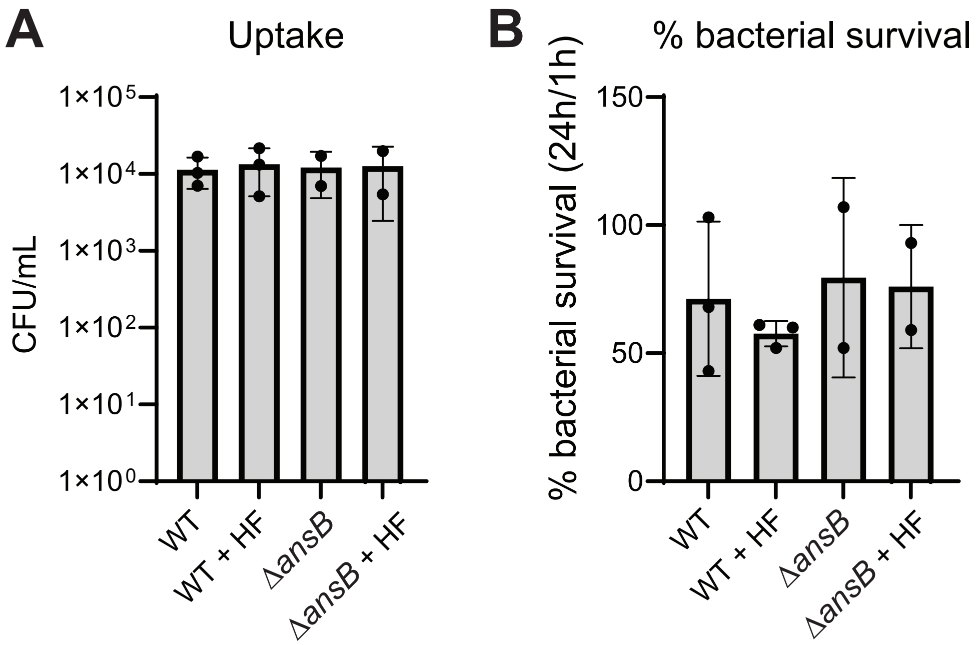

A
Uptake
CFU/mL
1×10^5
1×10^4
1×10^3
1×10^2
1×10^1
1×10^0
WT
WT + HF
ΔansB
ΔansB + HF
B
% bacterial survival
% bacterial survival (24h/1h)
150
100
50
0
WT
WT + HF
ΔansB
ΔansB + HF

**Supplemental Figure S3: Bacterial uptake and killing by U-937 cells with halofuginone treatment.** PMA-differentiated U-937 cells acclimated to D10 media were infected with *Salmonella* at MOI 10 +/- 80 nM halofuginone for the first 2h of infection to induce phospho-GCN2. **(A)** Cells were lysed at 1h pi and plated for CFU to measure uptake of WT and *ΔansB S.* Typhi and **(B)** 24hpi survival. n≥2.

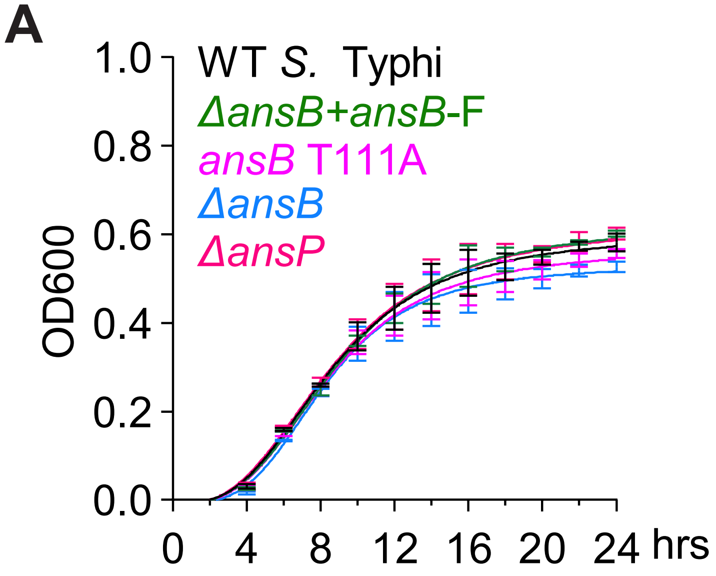
A
1.0
0.8
0.6
0.4
0.2
0.0
OD600
0
4
8
12
16
20
24
hrs
WT *S.* Typhi
*ΔansB*+*ansB*-F
*ansB* T111A
*ΔansB*
*ΔansP*

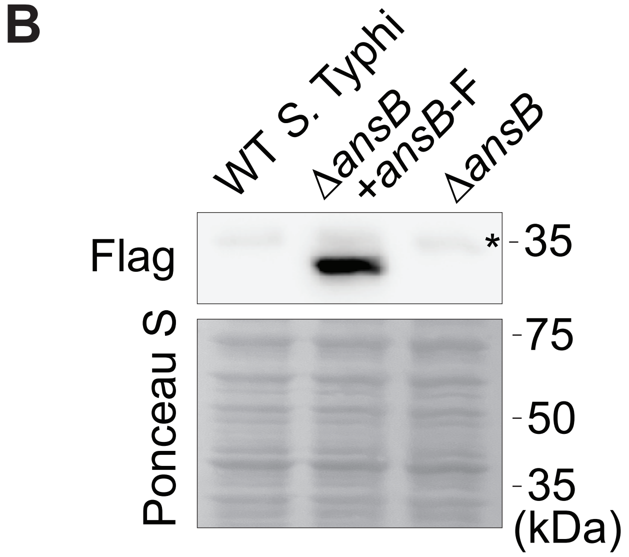
B
WT S. Typhi
*ΔansB* +*ansB*-F
*ΔansB*
Flag
Ponceau S
*
35
75
50
35
(kDa)

**Supplemental Figure S4: *In vitro* analysis of *S.* Typhi wildtype Ty2 (parent) and mutant strains. (A)** $10^5$ bacteria were inoculated in 150 µl LB, and their growth was monitored by spectrophotometer. **(B)** Fifty µL of overnight culture in LB was transferred to 2 mL of LB, and the bacteria were harvested when the OD reached 1.0. Ponceau S was used as a loading control. Asterisk indicates a non-specific band.

A

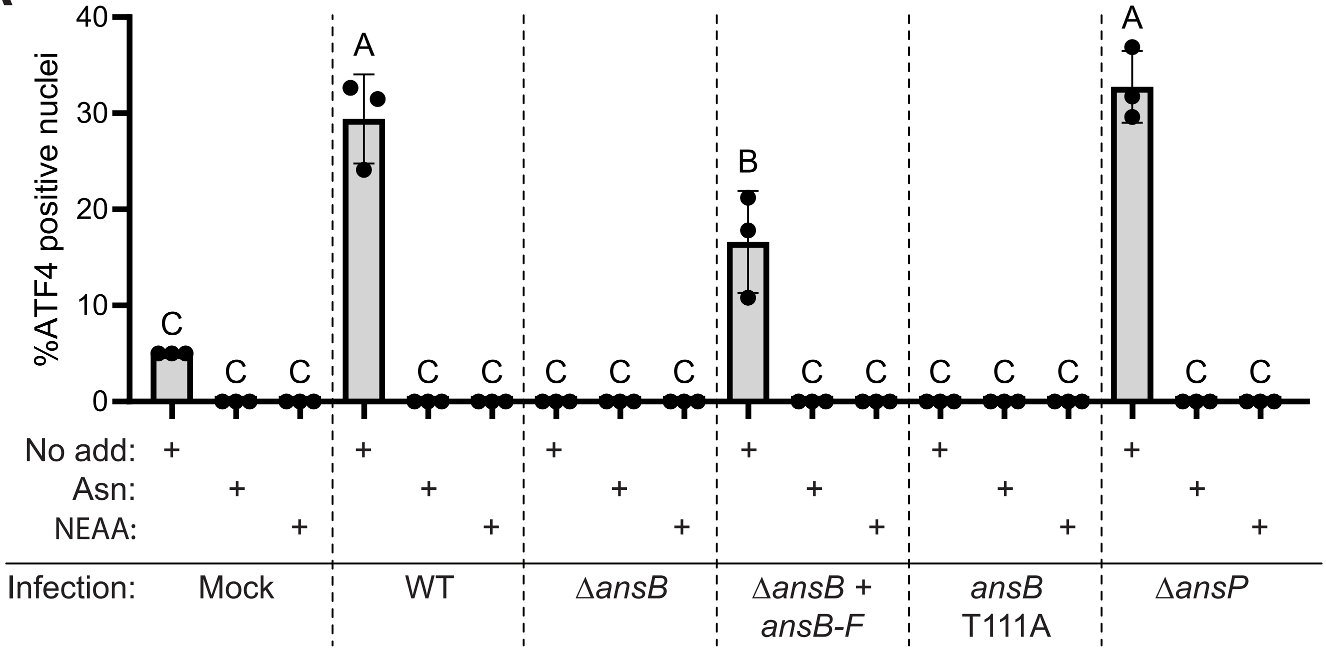
%ATF4 positive nuclei
40
30
20
10
0
C
A
B
A
C
No add:
Asn:
NEAA:
Infection:
Mock
WT
ΔansB
ΔansB + ansB-F
ansB T111A
ΔansP

**Supplemental Figure S5: Extended data associated with Figure 4B.** Extended data from Figure 4B (no add conditions) with Asn or NEAA supplementation. Murine BMDMs infected with MOI 10 with *S*. Typhi L-asparaginase II (*ansB*) mutants or asparagine transporter (*ansP*) imaged by automated confocal immunofluorescence microscopy at 8h pi. Quantification of three experimental replicates with points representing means of n=3 experiments with >1000 nuclei per condition per replicate. Unpaired one-way ANOVA and Tukey's post-test comparing column means with compact letter display statistical representation of comparisons with p-value < 0.05 (95). Error bars represent SD.

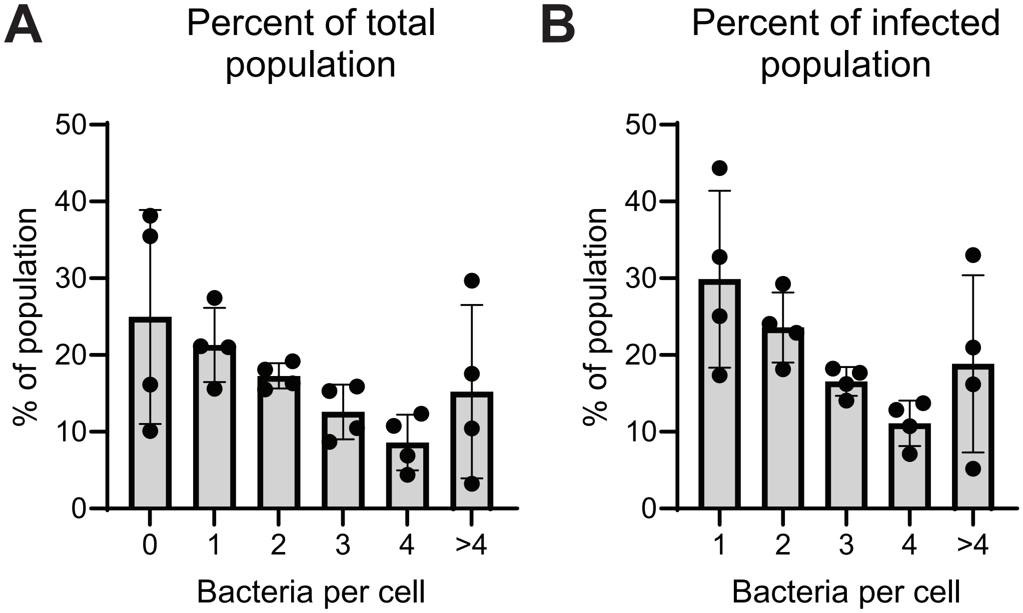

A
Percent of total population
% of population
50
40
30
20
10
0
0
1
2
3
4
>4
Bacteria per cell
B
Percent of infected population
% of population
50
40
30
20
10
0
1
2
3
4
>4
Bacteria per cell

**Supplemental Figure S6: Analysis of *S.* Typhi infection efficiency in BMDMs.** WT BMDMs infected for 8h with MOI 10 DsRed-expressing WT *S.* Typhi imaged by automated confocal immunofluorescence microscopy. **(A)** Number of bacteria per cell were quantified and data represented as a percentage of the total population or **(B)** as a percentage of the infected cell subpopulation.

**Supplemental table 1: Amino acids and concentrations used**

| **Essential amino acids (EAA) (Gibco #11130)** | **Stock concentration (mM) (50x)** |
|---|---|
| L-Arginine hydrochloride | 29.952606 |
| L-Cystine | 5.0 |
| L-Histidine hydrochloride-$H_2O$ | 10.0 |
| L-Isoleucine | 20.0 |
| L-Leucine | 20.0 |
| L-Lysine hydrochloride | 19.808743 |
| L-Methionine | 5.067114 |
| L-Phenylalanine | 10.0 |
| L-Threonine | 20.0 |
| L-Tryptophan | 2.5 |
| L-Tyrosine | 9.944752 |
| L-Valine | 20.0 |
| | |
| **Nonessential amino acids (NEAA) (Gibco #11140)** | **Stock concentration (mM) (100x)** |
| Glycine | 10 |
| L-Alanine | 10 |

| | |
|---|---|
| L-Asparagine | 10 |
| L-Aspartic acid | 10 |
| L-Glutamic Acid | 10 |
| L-Proline | 10 |
| L-Serine | 10 |

**Supplemental table 2: amino acid formulation in Gibco DMEM #12430054**

| Amino acids | Molecular weight | Concentration (mg/L) | mM |
|---|---|---|---|
| Glycine | 75.0 | 30.0 | 0.4 |
| L-Arginine hydrochloride | 211.0 | 84.0 | 0.39810428 |
| L-Cystine 2HCl | 313.0 | 63.0 | 0.20127796 |
| L-Glutamine | 146.0 | 584.0 | 4.0 |
| L-Histidine hydrochloride-H2O | 210.0 | 42.0 | 0.2 |
| L-Isoleucine | 131.0 | 105.0 | 0.8015267 |
| L-Leucine | 131.0 | 105.0 | 0.8015267 |
| L-Lysine hydrochloride | 183.0 | 146.0 | 0.7978142 |
| L-Methionine | 149.0 | 30.0 | 0.20134228 |
| L-Phenylalanine | 165.0 | 66.0 | 0.4 |
| L-Serine | 105.0 | 42.0 | 0.4 |
| L-Threonine | 119.0 | 95.0 | 0.79831934 |
| L-Tryptophan | 204.0 | 16.0 | 0.078431375 |
| L-Tyrosine disodium salt dihydrate | 261.0 | 104.0 | 0.39846742 |
| L-Valine | 117.0 | 94.0 | 0.8034188 |
